# Telerehabilitation in children and adolescents with intellectual disability: a systematic review

**DOI:** 10.3389/fpsyt.2026.1855260

**Published:** 2026-06-11

**Authors:** Martina Micai, Letizia Gila, Angela Caruso, Daniela Morelli, Maria Grazia Totino, Giulia Balboni, Carmen Belacchi, Alessandra Colucci, Chiara Fantini, Tiziana Metitieri, Margherita Orsolini, Alessandra Rampazzi, Ciro Ruggerini, Cristiana Stefani, Marco Bertelli, Francesca Fulceri, Maria Luisa Scattoni

**Affiliations:** 1 National Center for Rare Diseases, Istituto Superiore di Sanità, Rome, Italy; 2 Fondazione Santa Lucia IRCCS, Rome, Italy; 3 Centro Ricerca e Cura (CRC) Research and Care Center of Rome, Rome, Italy; 4 University of Bologna, Bologna, Italy; 5 University of Urbino Carlo Bo, Urbino, Italy; 6 Azienda Sanitaria Locale (ASL) Bari, Bari, Italy; 7 Azienda Ospedaliero-Universitaria (AOU) Meyer IRCCS, Firenze, Italy; 8 Department of Developmental and Socialization Psychology, Sapienza University of Rome, Rome, Italy; 9 Progetto Crescere Social Cooperative, Reggio Emilia, Italy; 10 CREA - Research and Clinical Center - San Sebastiano Foundation, Misericordia di Firenze, Florence, Italy

**Keywords:** caregiver-mediated intervention, digital health interventions, intellectual and developmental disabilities, online therapy, remote intervention, service delivery models, telehealth, telemedicine

## Abstract

**Introduction:**

In recent years, telerehabilitation has been increasingly used to improve access to care for children and adolescents with intellectual disability (ID). However, the available evidence is still limited and highly heterogeneous. This systematic review aimed to evaluate the impact of telerehabilitation interventions on cognitive, behavioural, and functional outcomes in this population.

**Materials and methods:**

This systematic review was conducted in accordance with Preferred Reporting Items for Systematic Reviews and Meta-Analyses (PRISMA) guidelines and registered in International Prospective Register of Systematic Reviews (PROSPERO) (CRD420251005874). A comprehensive search of PubMed and Web of Science databases was performed from inception to March 12, 2026. Eligible studies included randomized controlled trials and observational studies investigating telerehabilitation interventions in children and adolescents (≤18 years) with ID.

**Results:**

A total of 28 studies involving 668 participants were included. Interventions encompassed a wide range of approaches, including parent-mediated programmes, cognitive training, behavioural interventions, and tele-coaching models. Across studies, telerehabilitation was generally associated with improvements in language and communication skills, challenging behaviours, executive functions, and motor outcomes. Parent-mediated and telehealth-delivered behavioural interventions showed evidence, especially in reducing externalizing behaviours and parental stress. Digital cognitive training programmes showed feasibility and short-term gains in working memory and attention, although long-term effects were less consistent. Interventions targeting lifestyle and mental health showed promising but preliminary results. However, studies differed substantially in design, intervention protocols, and outcome measures, along with frequent methodological limitations.

**Discussion:**

Telerehabilitation appears to be a feasible and potentially effective approach for supporting children and adolescents with ID, particularly when caregivers are actively involved. Larger and methodologically robust studies are needed to better define intervention characteristics and assess long-term outcomes, as well as on the development of hybrid care models integrating in-person and remote approaches.

**Systematic Review Registration:**

https://www.crd.york.ac.uk/prospero/, identifier CRD420251005874.

## 1 Introduction

Intellectual Disability (ID) is a neurodevelopmental condition characterized by deficits in intellectual functioning and adaptive behaviour, with onset during the developmental period. Individuals with ID may experience difficulties across multiple domains, including reasoning, problem-solving, planning, academic learning, and understanding abstract concepts (1). Difficulties are accompanied by impairments in adaptive behaviour, which involve the conceptual, social, and practical skills relevant for managing daily life (2, 3). The estimated global ID prevalence depends on diagnostic criteria adoption and socio-cultural contexts. In the United States, the estimated prevalence in children aged 5 to 9 years is 2.04% (1.29–2.80) (4). Causes are multiple, including genetic factors, prenatal or postnatal events, and environmental factors (5). Common genetic causes are Down syndrome, Fragile X syndrome, and other chromosomal abnormalities or genetic mutations, but in a significant number of cases the etiology of the condition remains unidentified. Across the lifespan, people with ID experience co-occurring conditions, such as epilepsy, sensory and mobility problems, obesity and metabolic disease, and high rates of psychiatric disorders (6–8) and challenging behaviours (9). A multidisciplinary approach integrating psychiatry, neurology, psychology, pedagogy, and social care is considered essential to improving the quality of life of individuals with ID and facilitating their social inclusion (10, 11). Within this context, telemedicine has gained increasing relevance to improve access to healthcare services. Its importance has been particularly highlighted in emergency situations, such as the COVID-19 pandemic, during which remote service delivery became essential to ensure continuity of care for vulnerable populations, including children and adolescents with ID (12). The World Health Organization defines telemedicine as “the delivery of health-care services in which distance is a critical factor, provided by all health professionals using information and communication technologies to exchange information for the diagnosis, treatment, and prevention of disease and injuries, in the interest of improving the health of individuals and their communities” (13). Telerehabilitation is, in fact, a branch of telemedicine and aims to provide rehabilitation services using telecommunications technologies (14). Its purpose is to deliver medical-rehabilitation care and improve communication between professionals and users, facilitating access to services to overcome barriers such as geographical distance, time, or costs (15). Telerehabilitation can be used by health and social care professionals for assessment, monitoring, prevention, intervention, supervision, training, and consultation (16). Over the past decade, digital interventions, including teleconsultations and telemonitoring have been increasingly applied to neurodevelopmental disorders. Even if literature on individuals with autism spectrum disorders (ASD) is growing (17), evidence targeting children and adolescents with ID remains limited and largely unexplored. Telerehabilitation in ID presents unique challenges such as the need for continuous caregiver mediation and tailoring digital intervention to specific cognitive and sensory profiles. From a theoretical perspective, telerehabilitation in ID cannot be conceptualized as a simple remote transfer of in-person interventions. Rather, it often relies on a triadic model involving the clinician, the caregiver, and the individual, where therapeutic processes are mediated through relational and contextual factors embedded in everyday environments. This distributed and mediated nature of intervention may represent both a key mechanism of effectiveness and a source of variability across studies. Research on telerehabilitation in this population is still limited and evolving. Scientific studies on telerehabilitation for individuals with ID are steadily increasing aiming to generate evidence that enhances access to rehabilitation services and reduces geographical and logistical barriers. Telerehabilitation platforms may be used to deliver remote educational programmes aimed at developing academic and practical skills in individuals with ID. However, the effectiveness of the intervention also relies on the adaptation of content to individual needs and the level of supervision provided by parents or teachers. Assistive technologies, including specialized applications and augmentative and alternative communication (AAC) software, can be integrated into telerehabilitation platforms to support individuals with ID. In the everyday context, digital assistive intervention may be used to facilitate learning and enhance personal autonomy. Telerehabilitation also provides specialists with the opportunity to remotely monitor the use of these technologies and offer real-time technical support. Despite the increasing adoption of telerehabilitation in neurodevelopmental disorders, several critical gaps remain in the literature on ID. First, existing studies are highly heterogeneous in terms of intervention models, outcome measures, and levels of caregiver involvement, limiting the comparability and generalizability of findings. Second, unlike other neurodevelopmental conditions, interventions in ID often rely on continuous environmental and interpersonal mediation, raising questions about how therapeutic processes are maintained and transferred in remote contexts. Third, there is still limited understanding of which components of telerehabilitation interventions are most effective, for whom, and under which conditions. Addressing these gaps is essential to inform the development of tailored, scalable, and clinically meaningful intervention models for this population. Synthesizing the available evidence may help clarify the effectiveness, feasibility, and limitations of telerehabilitation interventions in the ID population. Previous evidence syntheses have examined telehealth and telerehabilitation in pediatric populations, but their scope differs from the present review. Prior reviews focused on remote cognitive training in genetic syndromes or congenital brain conditions (18), broader pediatric neurological and neurodevelopmental disorders (19), ASD (20), cerebral palsy (21), or tele-occupational therapy across mixed disabilities (22). In contrast, the present review specifically examines telerehabilitation interventions for children and adolescents with intellectual disability across multiple functional domains, including cognitive, behavioural, communicative, psychosocial, motor, and lifestyle outcomes. Therefore, this systematic review aims to critically evaluate the current evidence on telerehabilitation interventions for children and adolescents with ID, while also identifying key intervention characteristics, underlying mechanisms, and contextual factors that may influence their feasibility and effectiveness in real-world settings.

## 2 Materials and methods

This review was reported in accordance with Preferred Reporting Items for Systematic Reviews and Meta-Analyses (PRISMA) 2020 guidelines (see PRISMA 2020 Checklist in **eTable 1** in **Supplementary Materials**). The present systematic review was registered in the PROSPERO International Prospective Register of Systematic Reviews (registration number: CRD420251005874), and no deviations from the protocol were made. The evidence review team comprised researchers with expertise in systematic reviews and meta-analyses in neurodevelopmental disorders, including diverse professional backgrounds (psychology, neuro- and psychomotor therapy, child neuropsychiatry, and neurobiology).

### 2.1 Literature search

A comprehensive literature search of the PubMed and Web of Science databases was carried out from inception to 12 March 2026. No language restriction was applied. A combination of free text search and subject heading search was conducted using terms related to ID as well as terms related to telerehabilitation. To ensure that all relevant studies were captured, the keyword set for ID was refined with input from expert clinicians (see **eMethods 1** in **Supplementary Materials**). Randomized controlled trials (RCTs), observational studies, and case-control studies were included. Inclusion criteria consist of (1) Population: Children and adolescents aged ≤18 years old with a diagnosis of ID, according to the DSM-5-TR, (2) Intervention: the use of telerehabilitation for assessment, treatment, or support, (3) Outcome: change in intervention’s targeted behaviours/symptoms and/or change in individual clinical functioning. Records were excluded if they were case reports, comments, editorials, and qualitative evidence. Studies primarily focused on cerebral palsy or acquired brain injury were excluded unless intellectual disability or developmental delay was explicitly part of the target population. This choice was made to avoid excessive clinical heterogeneity, as these conditions often involve different rehabilitation pathways and intervention goals. Systematic reviews and meta-analyses were used to check for additional references.

### 2.2 Selection process

**Figure 1:**
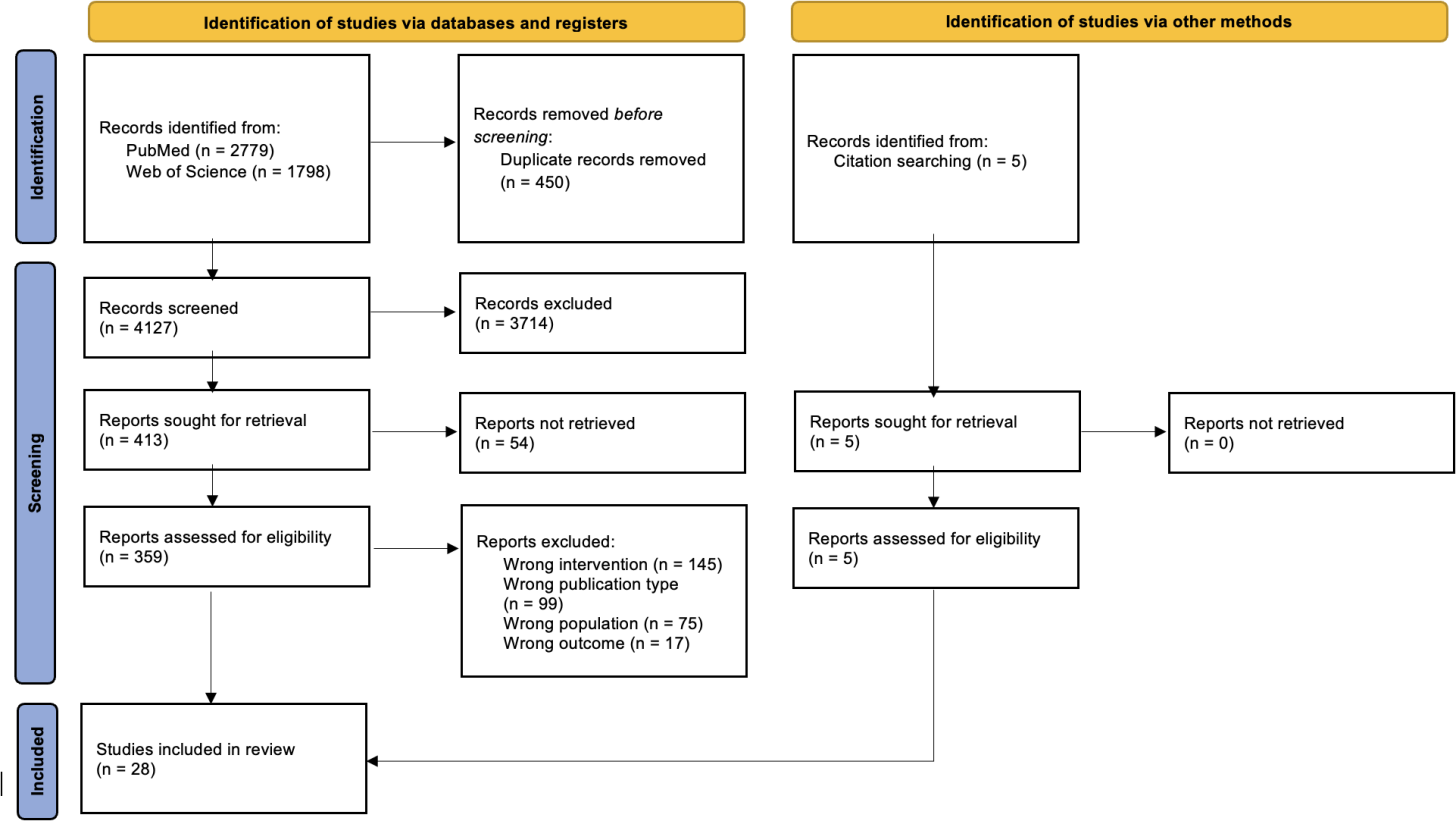
Flowchart of the literature selection process (24).

The reports retrieved from the search strategies were collected in the Systematic Review web app Rayyan QCRI (23). After removing duplicates, the screening and selection process was divided into three stages. First, titles and abstracts of each record retrieved were screened for inclusion and exclusion criteria by at least two authors (MM, LG, AC, and FF). The authors attributed the label “maybe” to the records that need to be checked for their full text. In the second stage, the full texts of the records with the label “maybe” were explored by the authors. Conflicts were discussed and, if necessary, the full texts were addressed independently by a third author. Finally, by checking the reference lists of the identified reviews, a hand search for additional eligible literature was conducted. A flow chart showing details of studies included and excluded at each stage of the study selection process is provided in **Figure 1**.

### 2.3 Data collection process

Data were extracted using an *ad hoc* data extraction form. To ensure consistency across all authors, calibration exercises were conducted before extracting the data. The following information was collected: (1) study characteristics: author, title, year of publication, source of funding, conflict of interest, and study design; (2) sample characteristics: country of recruitment, sample size, sex (number of females), age (mean, standard deviation, and range), and type of diagnosis; (3) type and description of the technology used, personnel involved (clinic-based professional, trained operator, parent/caregiver), and carer involvement when specified, and (4) addressed outcome domains with main findings regarding clinical and feasibility outcomes. A narrative approach was used to summarize the data collected into tables or visual synthesis. No meta-analysis was conducted due to the heterogeneity of study designs, populations, outcomes, and methodologies among the included studies.

### 2.4 Study quality

The methodological quality of each study was independently appraised by two blinded reviewers using the Mixed Methods Appraisal Tool (MMAT, 25). The MMAT is a comprehensive critical appraisal instrument designed for systematic reviews that incorporates diverse study designs, including qualitative, quantitative RCTs, quantitative non-randomized, quantitative descriptive, and mixed-methods studies. Any disagreements between the two reviewers were solved through consensus or by consulting a third blinded reviewer.

## 3 Results

The search strategy provided 4,577 studies from the following databases: PubMed (n = 2,779), Web of Science (n = 1,798), and hand searching of systematic reviews’ reference lists (n = 5). 450 duplicates were removed. A total of 4,127 records were screened for inclusion and exclusion criteria. Based on the titles and abstracts screening, 3,714 non-pertinent works were excluded. 54 reports were not retrieved after a reasonable time (1 month) had passed following contact with the authors.

The remaining 359 records were checked in their full text for the following reasons: 145 studies did not evaluate telerehabilitation as an intervention (wrong intervention), 99 studies were case reports, commentaries, editorials, qualitative evidence, or systematic reviews (wrong publication type), 75 studies did not include children and adolescents with ID (wrong population), and 17 studies did not report efficacy measures (wrong outcome). The systematic search from electronic databases resulted in the inclusion of 23 studies (26–48). Hand searching identified five additional studies that met the inclusion criteria and were included in the review (49–53). **Figure 1** illustrates the records identification and screening process.

### 3.1 Study and population characteristics

A total of 28 studies were included in this systematic review, encompassing around 668 children and adolescents (aged 0 to 18 years) diagnosed with ID. Some publications may represent multiple reports of the same underlying study. To minimize potential double-counting, the number of participants was extracted and described once. This may apply to Dimitropoulos (2021/2024), Hall (2020/2022), Kirk (2016/2017), and Verberg (2021/2022).

Publications were sparse between 2012 and 2016, followed by a gradual increase from 2017 onward, with a marked rise peaking in 2022 and sustained high output through 2024–2025. **eFigure 1** in **Supplementary Materials** shows the number of studies published each year from 2012 to 2026. All studies were published in English. Twenty of 28 studies (71%) reported no competing interests. Two studies (7%) did not report information on competing interests (i.e., no declaration or no competing-interest section), while six studies (21%) did. Twenty-three of 28 studies (82%) reported a funding source. Three studies (11%) reported no funding, while two studies (7%) did not report information on funding (i.e., no funding statement or the section was not provided). Regarding study design, nearly half of the studies were randomized controlled trials (RCTs; n = 13, 46.4%), followed by non-randomized studies (n = 9, 32.1%), quasi-experimental designs (n = 5, 17.9%), and one mixed-methods study (n = 1, 3.6%).

The studies included in the systematic review were predominantly conducted in Western countries, with 13 studies in North America (11 in the United States, 1 in Canada, and 1 across the United States and Canada), 8 in Europe (Italy, n = 3; the Netherlands, n = 3; Ireland, n = 1; Norway/Sweden, n = 1), and 4 in Australia, while the remaining studies were conducted in Asia (Hong Kong, n = 1; China, n = 1) and South America (Chile, n = 1).

Across the studies reporting sex distribution, the proportion of female participants varied markedly, ranging from 0% to 100%. Among the 25 studies with available data, 11 (44%) included less than 50% females; of these, 5 studies included no female participants (all involving fragile X syndrome samples). Two studies showed approximately 50% female, while 12 studies (48%) reported a female predominance (>50% female). Most studies involved mixed age groups spanning multiple developmental stages. Specifically, 8 studies (29%) included both school-age and adolescent participants, while 3 studies (11%) included preschool and school-age children, and 1 study (4%) covered early childhood through school age. Studies focusing on a single age group were less common: 6 studies (21%) targeted adolescents only, 5 (18%) focused on school-age children, and 4 (14%) on preschool children. Only 1 study (4%) included early childhood and preschool populations. With respect to diagnostic groups, all included samples comprised individuals with ID or DD. In addition, several studies included participants with specific genetic or syndromic conditions, such as Fragile X syndrome (n = 6, 21.4%), Prader–Willi syndrome (n = 4, 14.3%), and Down syndrome (n = 4, 14.3%). **Table 1** summarizes the main methodological and clinical characteristics of the included studies, while **eTable 2** provides a more detailed description of study design, sample features, interventions, and outcomes.

**Table 1:** Included studies organized by domain, diagnosis, age group, and study design.

Domain	Diagnosis	Age group	Study	Study design
Challenging behaviour	DD + externalizing behaviour problems	PS	Bagner et al. (26)	RCT
	ID, DD (chromosomal/genetic syndromes, cerebral palsy)	EC + PS + SA	Grenier-Martin et al. (34)	RCT
	FXS	PS + SA	Hall et al. (35)	RCT
	FXS	SA	Hall et al. (36)	Non RCT and RCT
	FXS; ID + frequent comorbid autism features	AD	Miranda et al. (42)	QD
	DD, with and without ASD and other medical/genetic or speech/language delays	PS	Warner et al. (47)	RCT
Language, communication, and social play	PWS (genetically confirmed)	SA	Dimitropoulos et al. (29)	Non RCT
	PWS (mUPD and DEL subtypes)	PS	Dimitropoulos et al. (30)	Non RCT
	PWS; social-cognitive and adaptive impairments	SA	Dimitropoulos et al. (31)	Non RCT
	Developmental Language Disorder/DS	EC + PS	Frizelle et al. (32)	MM
	ID	SA	Li et al. (40)	QD
	FXS	SA + AD	McDuffie et al. (41)	RCT
	FXS	SA + AD	Nelson et al. (44)	RCT
	PWS	PS	Zyga et al. (48)	QD
Cognitive skill	FXS, ID	SA + AD	Hessl et al. (49)	RCT
	ID, DD	PS + SA	Kirk et al. (50)	RCT
	ID, DD, ASD, DS, nonspecific ID, and other genetic disorders	PS + SA	Kirk et al. (51)	RCT
	DS	SA + AD	Pulina et al. (52)	Non RCT
	ID with and without comorbid conditions	SA + AD	Söderqvist et al. (53)	RCT
Mental health	DD, DD secondary to neurological conditions	SA	Bompard et al. (27)	Non RCT
	Borderline, mild, and moderate ID + anxiety symptoms	AD	Hronis et al. (37)	QD
	Mild to borderline ID	AD	Verberg et al. (45)	RCT
	Mild to borderline ID	AD	Verberg et al. (46)	RCT
Lifestyle	ID (DS, DeGeorge syndrome, chromosomal micro-deletion, unspecified ID)	AD	Curtin et al. (28)	Non RCT
	DS	SA + AD	Giuriato et al. (33)	Non RCT
	Mild to moderate ID	AD	Kiewik et al. (38)	Non RCT
	Mild ID + overweight/obesity	SA + AD	Lee et al. (39)	RCT
Reading	DS	SA + AD	Murphy et al. (43)	QD

AD, Adolescence; ASD, Autism Spectrum Disorder; DD, Developmental Delay; DS, Down Syndrome; EC, Early Childhood; FXS, Fragile X Syndrome; ID, Intellectual Disability; MM, Mixed Methods; PS, Preschool; PWS, Prader–Willi Syndrome; QD, Quantitative Descriptive; SA, School Age. Study design classifications were harmonized from MMAT categories reported in **eTable 2**.

### 3.2 Quality assessment

The included studies were predominantly RCTs (15 studies, 54%). Non-randomized quantitative studies represented 9 studies (32%), while quantitative descriptive studies accounted for 5 studies (18%). One study used a mixed-methods approach (36), combining quantitative and qualitative methods. Overall, the methodological quality of the included studies showed substantial variability across designs and MMAT domains. Detailed scores for each study and MMAT item are provided in **eTable 2**. All studies met the initial screening criteria (clear research questions and data adequate to address them), but their performance across the five methodological dimensions differed considerably depending on study type. Stronger methodological quality was observed in RCTs, while non-randomized designs showed moderate quality and quantitative descriptive studies showed moderate-to-low quality. The mixed-methods study showed high quality in its qualitative component but weaker methodological rigor in its randomized component.

### 3.3 Target domains of telerehabilitation

**Figure 2:**
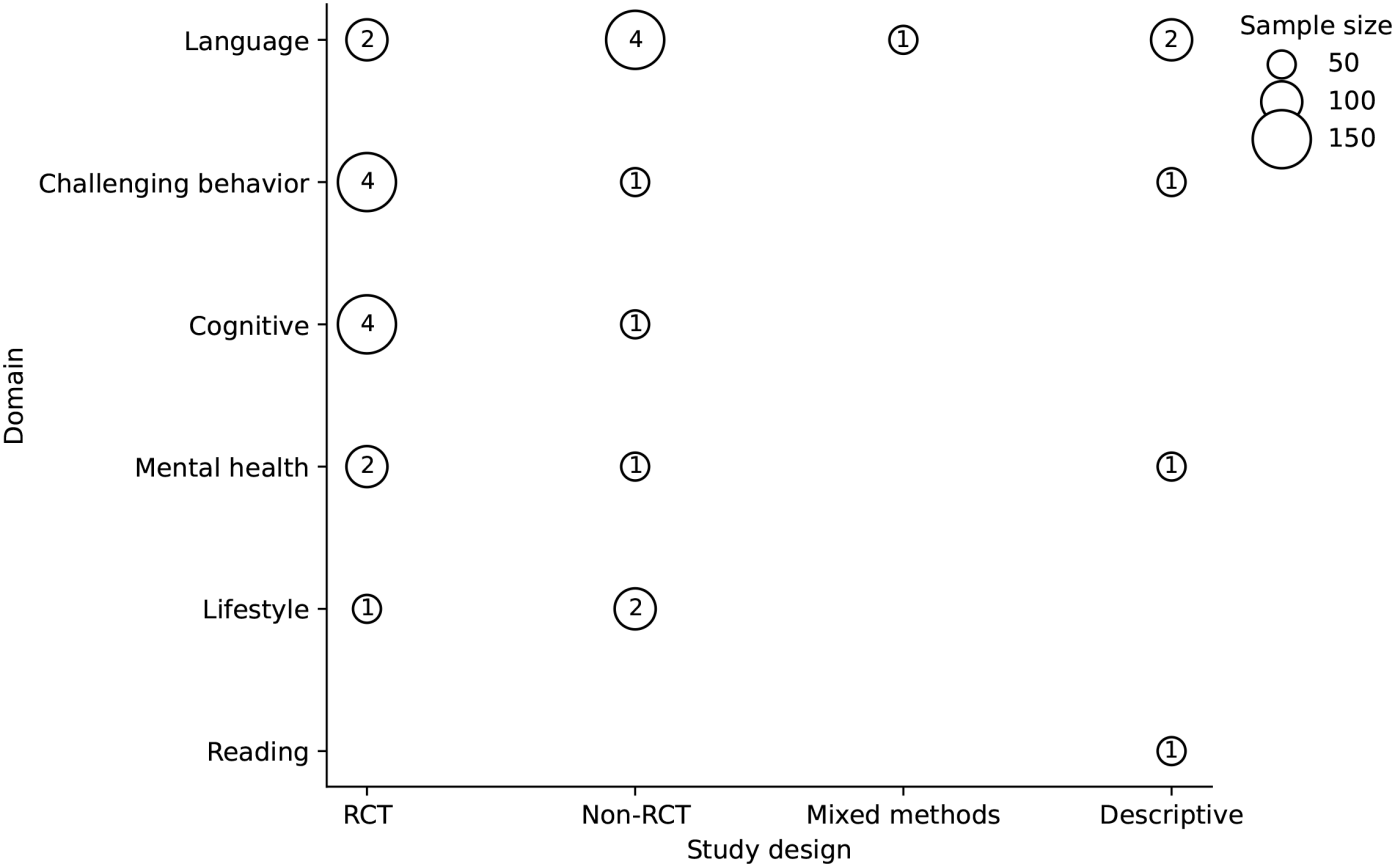
Distribution of studies across intervention domains and study designs. Numbers inside the bubbles represent the number of studies for each domain and study design. Bubble size represents the total sample size, calculated as the sum of participants across the studies included in each corresponding category.

**Figure 3:**
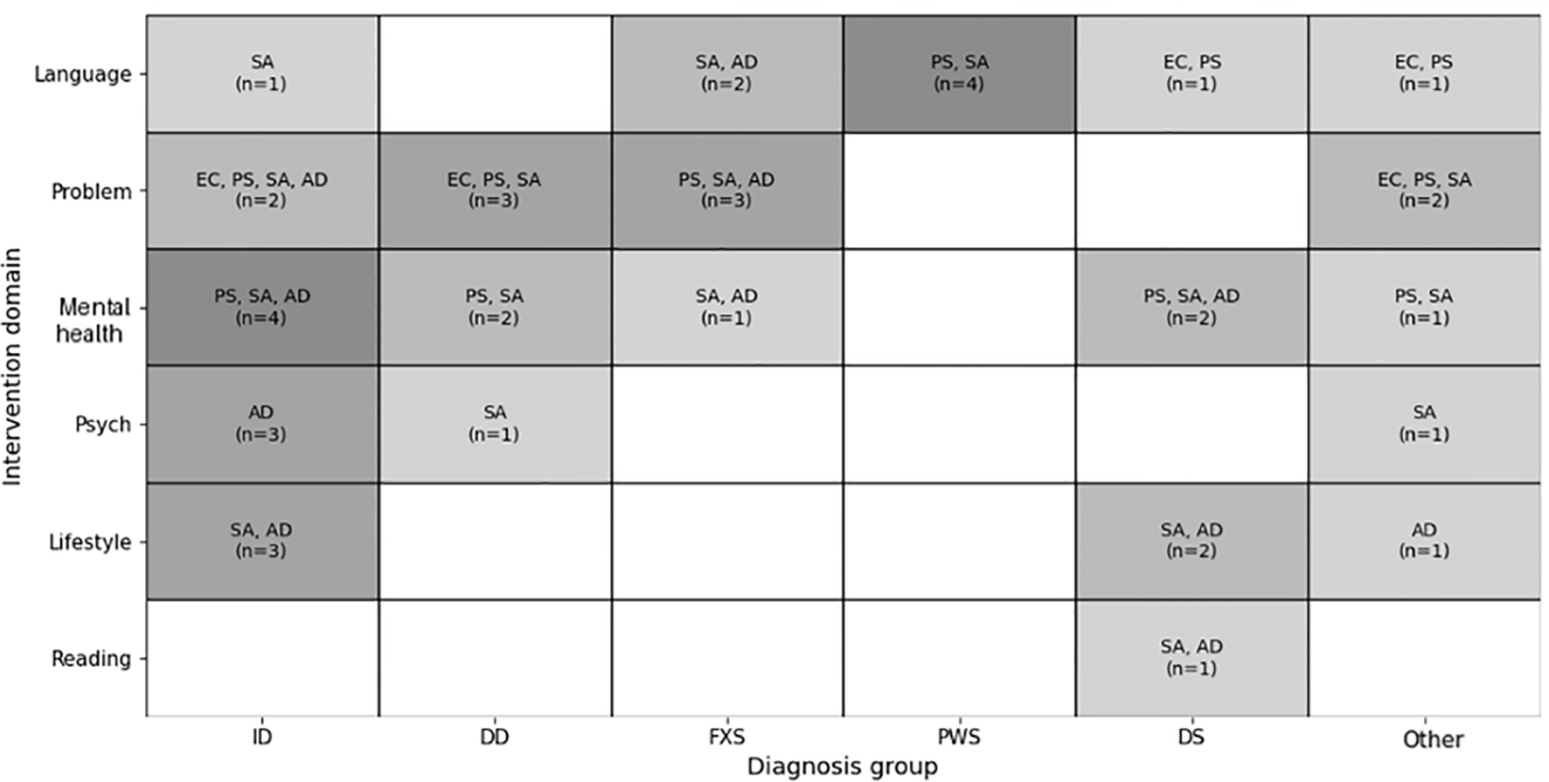
Clinical and age profile of population explored across intervention domains. Cells report age groups represented in each domain-diagnosis combination; darker shading indicates more studies. Diagnostic categories are not mutually exclusive because some studies included mixed samples. AD, Adolescence; DD, Developmental Delay; DS, Down Syndrome; EC, Early Childhood; FXS, Fragile X Syndrome; ID, Intellectual Disability; PS, Preschool; PWS, Prader–Willi Syndrome; SA, School Age. Diagnostic categories were grouped for synthesis purposes, while detailed diagnoses are reported in **eTable 2**. Detailed age distributions (means and ranges) are reported in **eTable 2**.

In this review, telerehabilitation was operationally defined as including synchronous videoconferencing interventions, parent-mediated tele-coaching models, asynchronous web-based programmes, computerized cognitive training, or blended interventions combining remote and in-person components. The heterogeneity of the included interventions reflects the multidimensional rehabilitation needs of children and adolescents with intellectual disability. To address this variability, findings were synthesized narratively and grouped by intervention domain. Therefore, conclusions should be interpreted as domain-specific rather than as general statements applicable to all telerehabilitation interventions. The following target domains of telerehabilitation were classified according to the primary aim of the intervention and its main outcomes assessed, with emphasis on the functional area most directly addressed. Studies most frequently targeted language, communication, and social play (n = 8, 28.6%), followed by challenging behaviours (n = 6, 21.4%), cognitive skills (n = 5, 17.9%), mental health outcomes (n = 4, 14.3%), and lifestyle (n = 4, 14.3%), while only one study addressed reading outcomes (n = 1, 3.6%). To enhance clinical interpretability, results were summarized using an evidence mapping approach, visually representing the number of studies included for each type of studies across domains (**Figures 2**, **3**).

#### 3.3.1 Language, communication, and social play

Eight included studies investigated telerehabilitation in the areas of language, communication, and social play. One study by McDuffie et al. (41) explored the effectiveness of an intervention on the development of narrative language through coaching on maternal responsiveness in a group of 20 children with Fragile X, aged 10 to 17 years. The authors, based on parent-child interactions focused on sharing a picture book, taught mothers to use the following strategies: making comments related to the child’s expressions about the story, asking open-ended questions (Who? When? Where?), and using sentence completion prompts to support the child, expanding on their responses. The intervention, developed over 12 weeks, included an initial support session conducted by a behavioural analyst, followed by a parent training session on the techniques. After that, each week, a synchronous therapy session was first conducted, where clinicians provided immediate feedback. Then, videos were sent to be reviewed in video feedback sessions, followed by an observational session of the interaction. The entire intervention was delivered remotely. The analysis of the recordings of the interactions between the children/adolescents and their mothers (at home and in the clinic) and, subsequently, with a neutral examiner, revealed that the mothers learned the strategies, showed increased involvement in the interaction, and the children developed their lexical skills. However, no significant results were found regarding the development of grammatical skills or the generalization of the child’s involvement in the storytelling sessions with the examiner. The study has some limitations, including the fact that the intensity of the intervention was not homogeneous across dyads, the fidelity of implementation was not measured, and there was no follow-up.

A study by Nelson et al. (44) provides preliminary evidence that participation in a parent-implemented language intervention delivered remotely, situated within the context of shared storytelling, can lead to an increase in the use of inferential language by 19 children and adolescents aged 10 to 17 years with Fragile X. The mother-child dyads were randomly assigned to either an intervention group or a comparison group. The materials used included about 30 illustrated children’s books, which were modified by removing the accompanying text on each page. For each book, written scripts were created for the mothers that summarized the key events of the story; these scripts also included examples of questions that could be asked to the children/adolescents. Participants in the treatment group showed a marked increase in their use of prompted inferential language, but not spontaneous language, during the post-intervention sessions. This study provides initial support for the usefulness of a parent-implemented language intervention to increase the use of inferential language by school-age children with Fragile X but also suggests the need for additional treatment to encourage spontaneous use (44).

The aim of some studies focused on children with Prader-Willi syndrome was to train parents in supporting their children’s pretend play through remote training (29, 30, 48). The programme included a preliminary in-person phase, which involved an assessment of the child and observations of parent-child play interactions. In an intervention phase lasting 6 weeks (with two sessions per week) or 8 weeks (with slightly longer sessions but only once a week), parents were trained in four main areas: a) engagement and play; b) managing dysfunctional behaviours; c) understanding emotions and coping skills; d) social skills and peer interactions. An analysis of parents’ perception of the programme showed high levels of acceptability and satisfaction (48).

An analysis of the effectiveness of the intervention (30) showed that improvements in post-intervention measures occurred only when children were involved in the parents’ training sessions (with periodic monitoring by an online operator), with significant differences observed compared to the control group. Specifically, significant improvements were noted in the children’s ability to organize play, as well as in the frequency of pretend play behaviours and emotional expression. However, these improvements were only observed in the subgroup of children with the genetic variant of maternal uniparental disomy. In the subgroup with a paternal chromosome deletion, no significant differences were found compared to the control group.

More recently, Dimitropoulos et al. (31) evaluated the efficacy of a refined remote, play-based PRETEND programme for school-aged children with Prader–Willi syndrome using a waitlist-controlled design. The updated protocol increased parent involvement (including structured parent–child joint play sessions) and allowed greater individualization of play goals to match each child’s needs. Children in the intervention group showed significant improvements in the organization of pretend play (with a large effect), alongside positive patterns of change in affect expression and thematic content, whereas these changes were not observed in the waitlist control group. The authors also reported that several children reached levels comparable to, or exceeding, reference values on core pretend-play variables after the intervention.

Frizelle et al. (32) investigated the feasibility and acceptability of an online “language-through-music” programme for very young children with Down syndrome (approximately 12–42 months), delivered at home and designed to support vocabulary development by combining singing, everyday objects, and key word signing. In the quantitative phase, 76 families were randomly assigned to a high-frequency (five times/week) or low-frequency (twice/week) dosage condition. Vocabulary outcomes were measured through parent-report checklists capturing receptive vocabulary and different expressive modalities (e.g., signing, word imitation, and spontaneous word use). Overall analyses did not show an added benefit of the higher dosage compared with the lower dosage; however, exploratory models suggested that children with stronger baseline language skills might gain more from the higher-frequency condition. Parents reported the programme as positive and acceptable for the family.

Overall, these studies suggest that telerehabilitation interventions targeting language, communication, and social play are feasible and generally well accepted by families, particularly when based on structured parent-mediated approaches. Improvements are most consistently observed in specific trained skills, such as lexical development, inferential language, and play organization, whereas evidence for the generalization of these gains across contexts and for broader language domains remains limited.

#### 3.3.2 Reading

In a pilot study conducted during the COVID-19 pandemic, Murphy et al. (43) used an interactive web application to promote reading and spelling skills in 6 Australian children with Down syndrome, aged between 8 years and 6 months and 12 years and 9 months. The training lasted 6 weeks with three 60-minute sessions per week and included word-related activities (e.g., phonemic awareness, high-frequency word recognition, spelling skills), reading fluency exercises, tasks for comprehension of interactive stories, and story comprehension activities managed by the parents. Parents received a training session to teach them how to alternate with the child in reading aloud, ask a question for each page read, and support self-correction of decoding errors. Parents had access to online resources guiding how to manage the activities and were involved in providing a summary of the activities carried out in each of the 12 shared story comprehension sessions. The results of the study showed a statistically significant improvement in the children’s reading accuracy and fluency. However, no significant improvement was reported in text comprehension. Evidence on reading outcomes is currently limited to a small number of studies, with preliminary findings suggesting improvements in reading accuracy and fluency, but not in comprehension. These results should be interpreted with caution given the limited evidence base.

#### 3.3.3 Mental health

Bompard et al. (27) conducted a pilot study evaluating the remote delivery of a music “therapy” programme based on the Euterpe method over a 12-day period. The intervention involved 14 children with developmental delay and relied on a personalized soundtrack combined with multimodal sensory stimulation, including visual stimulation through coloured lights placed in the therapy room, olfactory stimulation via aroma diffusers, and tactile stimulation using a tuning fork and vibrating devices. The stimulation parameters could be adapted to participants’ individual needs and specific therapeutic targets. Results indicated a significant improvement in children’s sleep quality and a reduction in parental stress.

Verberg et al. (45) evaluated the “The Growth Factory program” in a RCT involving 46 adolescents with ID, aged <15 years (overall sample: 119 participants aged 12–23 years). No subgroup-specific results were reported for participants under 15 years; intervention effects were consistent across age groups, with no moderating effect of age. The study aimed to improve mindset, treatment adherence, and psychosocial development, particularly in terms of self-esteem and mental health. The Growth Factory is an online mindset intervention designed for youth with mild to borderline ID, combining psychoeducation on brain plasticity and growth mindset with interactive exercises, video-based peer role models, and cognitive-behavioural strategies. The intervention comprised six online sessions delivered over six weeks, followed by booster sessions at three months for the experimental group in addition to treatment as usual, while the control group received treatment as usual only. Both groups underwent pre- and post-intervention assessments, as well as two follow-up assessments at 3 and 6 months. The results indicated high participant satisfaction and demonstrated the effectiveness of the programme in improving mindset, self-esteem, and mental health outcomes. These effects were maintained at the 6-month follow-up, except for mental health problems, for which improvements were sustained only up to 3 months. No significant effects were observed for empowerment, externalizing problems, treatment motivation, or therapeutic alliance.

Verberg et al. (46) further investigated the mediators and moderators of the “The Growth Factory program”. Overall sample: 119 participants aged 12–23 years. As for the previous study, no subgroup-specific results were reported for participants under 15 years; intervention effects were consistent across age groups, with no moderating effect of age. Specifically, the authors examined mindset, gender, age, level of ID, and intervention satisfaction as potential mediators and moderators of the outcomes reported in the previous trial (45). The findings suggested that treatment effectiveness was independent of age and cognitive level, and that participants reporting higher satisfaction showed greater improvements in both internalizing and externalizing mental health problems. Moreover, the intervention was more effective in reducing internalizing symptoms among girls and in increasing perseverance among boys, which in turn indirectly mediated both internalizing and externalizing outcomes at the 3-month follow-up.

Finally, Hronis et al. (37) showed the feasibility of a cognitive–behavioural therapy programme, “Fearless Me!”, aimed at reducing anxiety symptoms through face-to-face sessions supplemented by online exercises. The programme was tested in a sample of 21 adolescent girls with ID (age range: 12–18 years). The results indicated full intervention completion despite partial non-completion of some online components and showed an overall reduction in anxiety symptoms as reported by both self-assessments and teacher ratings.

Taken together, telerehabilitation interventions targeting psychological and psychosocial outcomes show promising results in improving aspects such as sleep quality, self-esteem, mindset, and anxiety symptoms, along with high levels of participant satisfaction. However, these findings are primarily derived from studies with small sample sizes, heterogeneous designs, and, in some cases, limited reporting of subgroup-specific outcomes. As a result, the strength of the evidence remains preliminary, and further methodologically robust studies are needed to confirm these effects and clarify their clinical significance.

#### 3.3.4 Cognitive skills

Hessl et al. (49) evaluated the effectiveness of a working memory training programme using the *Cogmed* software, delivered via personal computers and tablets. Cogmed is a computerized working memory training programme based on repeated visuospatial and auditory memory tasks, with training intensity tailored to the participant’s performance level and developmental profile. This double-blind RCT included two groups of participants with Fragile X syndrome, involving 100 children and adolescents (37 females, 63 males; mean age = 15.2 years). The intervention group completed 20–25 sessions over 5–6 weeks with task difficulty adaptively adjusted to individual performance, whereas the control group received the same training at a non-adaptive difficulty level. Training activities comprised visuospatial and auditory working memory tasks, supported by parents and calibrated to participants’ functional levels. Treatment adherence was high, with 100% session completion in the experimental group and 98% in the control group. Outcomes were assessed at baseline, post-intervention, and at 3-month follow-up using standardized executive function and attention measures, as well as parent- and teacher-reported questionnaires. Both groups showed significant improvements in working memory that were partially maintained at follow-up, with no significant between-group differences, suggesting that adaptive difficulty did not confer additional benefit beyond repeated practice.

Kirk et al. (50) conducted a double-blind RCT examining the effectiveness of the computerized attention training programme *Training Attention and Learning Initiative (TALI)* in 76 children with ID (IQ < 70) and attention difficulties, aged 4–11 years. The intervention consisted of home-based training on a touchscreen tablet, delivered in 20-minute daily sessions for five weeks, targeting selective attention, sustained attention, and attentional control. The experimental group received adaptively adjusted tasks, whereas the control group completed non-adaptive versions with minimal attentional demands; both groups used interactive guides and reward systems to support motivation. Adherence was higher in the intervention group (90%) than in the control group (70%). Results indicated that the intervention group showed significant improvements in selective attention, which were maintained at the 3-month follow-up, while no significant effects were observed for sustained attention or attentional control. Parent- and teacher-reported hyperactivity decreased in both groups post-intervention and at follow-up, with no significant between-group differences.

In a subsequent study, Kirk et al. (51) assessed the generalization of TALI training effects to non-trained domains, including academic skills, executive functioning, and behaviour, in the same cohort of 76 children following identical intervention procedures. Both groups completed 25 daily sessions and were assessed at baseline, post-intervention, and at 3-month follow-up. No significant between-group differences were observed in literacy, executive functions, or behavioural and emotional outcomes. However, the intervention group demonstrated a significant improvement in numeracy skills at follow-up, suggesting that intensive computerized attention training may transfer to selected untrained academic domains.

Pulina et al. (52) investigated the effectiveness of a computerized visuospatial working memory training programme in 39 children and adolescents with Down syndrome (23 females; mean age = 12 years and 5 months). The experimental group completed eight 30-minute sessions over one month under the supervision of a psychologist, whereas the control group completed the same exercises under parent supervision following appropriate training. Both groups engaged in identical activities targeting simultaneous working memory, spatial–sequential skills, visuospatial abilities, and memory. Improvements were observed in both groups and were maintained at 1-month follow-up, indicating that parent-delivered home-based training can be effective.

Finally, Söderqvist et al. (53) evaluated the feasibility and efficacy of a computerized cognitive training programme aimed at improving working memory and non-verbal reasoning in 41 children with ID (19 females; age range: 6–12.5 years) in a double-blind RCT. The experimental group (n = 22) received adaptively adjusted training, while the control group (n = 19) received non-adaptive training, across 25 sessions over five weeks, with parental or teacher supervision. Approximately 85% of participants completed an average of 24 sessions. Substantial interindividual variability in training progress was observed. Improvements generalized to verbal working memory and instruction comprehension, with progress predicted by gender, comorbidity status, and baseline verbal working memory. Girls and participants without comorbidities and with higher baseline performance showed greater gains. However, no significant improvements were maintained at the 1-year follow-up, and no effects were observed on behavioural outcomes or attentional symptomatology, suggesting that training intensity and repetition may be required to sustain benefits.

Overall, computerized training for cognitive skills interventions delivered via telerehabilitation demonstrate high feasibility and adherence, with participants and caregivers generally able to engage consistently with structured digital tasks. However, evidence for their effectiveness remains mixed, with improvements often limited to trained cognitive domains, such as working memory or selective attention, and limited generalization to broader functional outcomes. These findings suggest that repeated practice and task exposure may account for part of the observed gains, rather than specific intervention components.

#### 3.3.5 Challenging behaviour

Six of the included studies examined the use of telerehabilitation to address challenging behaviours. The RCT by Bagner et al. (26) demonstrated the effectiveness of a Parent–Child Interaction Therapy (PCIT) intervention delivered through 20 videoconference sessions, both in achieving a significant reduction in externalizing behavioural problems, maintained at the 1-year follow-up, and in increasing positive parenting behaviours. The study involved 75 children in the experimental group and 75 in the control group, with a mean age of 36.2 months and a diagnosis of developmental delay accompanied by clinically significant behavioural problems.

Warner et al. (47) conducted a secondary analysis focusing on sleep-related outcomes. Using path analyses in the full sample of 150 children with developmental delay (randomized to iPCIT vs referrals-as-usual), the authors found that iPCIT significantly reduced bedtime resistance behaviours post-treatment, and that these reductions were associated with better caregiver-reported sleep quality at the 6-month follow-up.

Hall et al. (35) evaluated the effectiveness of a telehealth-delivered behavioural intervention for children with Fragile X syndrome in a RCT. The 57 children recruited, aged 3–10 years, were assigned to an experimental group (tele-intervention) or a control group (treatment as usual) and participated over a 12-week period. The intervention included preliminary sessions for parents and required caregivers to support children in managing the technological tools. Child sessions were delivered via teleconferencing by Board Certified Behavior Analysts following a functional behavioural assessment. Pre- and post-intervention outcomes were assessed through direct observation of challenging behaviours and standardized measures. Results for the experimental group indicated significant reductions in irritability, stereotyped behaviours, and overall challenging behaviours, alongside decreased parental stress and high intervention acceptability. These benefits were maintained and further enhanced following a booster session approximately three years after training, as reported by Hall et al. (36) in a long-term follow-up conducted with a subsample of 24 participants who had received Functional Communication Training.

Miranda et al. (42) reported a brief, parent-implemented telehealth intervention for families of children with Fragile X syndrome in Chile, targeting parental stress and challenging behaviours. The programme was delivered fully online via commonly available platforms and consisted of four sessions provided every two weeks. Sessions were individualized, combining psychoeducation about the diagnosis, functional behaviour understanding (including the motivation behind target behaviours), and collaborative development of practical support strategies embedded in daily routines. Pre–post comparisons indicated reductions in parental stress and improvements in parents’ understanding of challenging behaviour and perceived parenting competence.

Grenier-Martin et al. (34) tested the effectiveness of an online intervention targeting the management of challenging behaviours in parents of children with ID in a RCT. Twenty-nine caregivers were allocated to an intervention group (16 families) or a waitlist control group (13 families). The children (18 males and 11 females) had a mean age of 4 years and 1 month and all had a confirmed diagnosis of ID, predominantly associated with genetic conditions. The intervention consisted of asynchronous online sessions for parents, totaling approximately 4 hours of training delivered across five modules addressing behavioural assessment, antecedent evaluation, prevention strategies, teaching appropriate behaviours, and a final synthesis module. Each module included a digital presentation, video instructions, and a downloadable written guide. Parents and children were assessed pre- and post-intervention. Following the intervention, the experimental group showed a significant reduction in both the frequency and severity of challenging behaviours compared with the control group. A significant reduction in parental stress was also observed post-intervention, although this effect was not maintained at follow-up, while parental self-efficacy improved significantly. Overall acceptability and parent-reported satisfaction were high.

Taken together, telerehabilitation interventions targeting challenging behaviours show consistent feasibility and promising effectiveness, particularly when based on parent-mediated and tele-coaching models. These approaches appear to be effective in reducing externalizing behaviours and parental stress, highlighting the central role of caregivers as active agents in the intervention process. However, variability in intervention protocols and outcome measures, along with limited long-term follow-up data, restricts the ability to draw firm conclusions about sustained effects.

#### 3.3.6 Lifestyle

Kiewik et al. (38) conducted a controlled pilot study aimed at improving knowledge and attitudes toward alcohol and smoking among adolescents with mild to moderate ID (aged 12–16 years; mean age = 14.72). The 35 participants in the experimental group who completed the online programme *“Prepared on Time”* did not show a significant increase in knowledge levels following the two-week intervention, compared with the 34 participants in the control group who received a standard educational programme.

Curtin et al. (28) assessed the feasibility and effectiveness of an online sports and food literacy programme. The intervention was delivered to six adolescents with ID (two females; mean age = 15.3 years) across 12 online sessions of 70 minutes each. Sessions focused on improving motor skills through verbal instruction, visual demonstration, and guided practice of motor actions (e.g., dribbling, jumping, throwing), as well as on food literacy through game-based lessons promoting healthy eating. The programme was delivered by a sports coach and a dietitian. Online assessments indicated improvements in motor performance, perceived motor competence, and motivation, alongside increased food knowledge and greater willingness to consume vegetables and drink water. No improvements were observed in the consumption of snacks and fruit. The programme was well accepted, and parental satisfaction was high, although only half of the participants reported enjoyment of the motor components.

Giuriato et al. (33) conducted a tele-coaching pilot study to enhance motor skills in 18 children and adolescents with Down syndrome through a remotely delivered, game-based exercise programme. The intervention was delivered via an online platform and consisted of 60-minute sessions, three times per week for 15 weeks, supervised by sports-science coaches in small groups (approximately one coach per three participants). Pre–post results indicated improvements in balance and a reduction in systolic blood pressure, while changes in body composition indices and parent-reported cognitive domains (executive function, memory, language) were not significant.

Finally, Lee et al. (39) implemented a blended intervention combining face-to-face training and teleconsultation, with parental involvement, aimed at promoting physical activity, healthy eating behaviours, and overall healthy lifestyles among students with mild ID (76% male; aged 8–16 years) recruited from special education schools over a 24-week period. The authors reported modest improvements in behaviour and self-image, as well as a slight reduction in overweight indices.

Overall, telerehabilitation interventions targeting lifestyle outcomes show promising feasibility and potential benefits in areas such as physical activity, weight management, and health-related behaviours. However, the limited number of studies, together with heterogeneity in intervention design and outcome measures, restricts the strength of the evidence and highlights the need for further research to establish their effectiveness and long-term impact.

## 4 Discussion

This systematic review highlights that telerehabilitation represents a feasible and increasingly adopted approach for supporting children and adolescents with ID, with evidence suggesting domain-specific benefits across behavioural, cognitive, and psychosocial outcomes. However, beyond these findings, a key insight emerging from the literature is that the effectiveness of telerehabilitation in this population appears to rely less on the simple remote delivery of interventions and more on the extent to which therapeutic processes are mediated by caregivers and embedded within everyday contexts. When considered across intervention domains, three main patterns emerge. First, interventions that actively involve caregivers, particularly through parent-mediated or tele-coaching approaches, tend to show more consistent and clinically meaningful effects, especially in the reduction of challenging behaviours and in the enhancement of communication and social interaction. Second, interventions primarily based on structured digital training, such as computerized cognitive programmes, show high feasibility and adherence but more limited and domain-specific effects, with scarce evidence of generalization to broader functional outcomes. Third, interventions targeting psychosocial and lifestyle domains show promising but still preliminary results, often constrained by small sample sizes, heterogeneous designs, and limited long-term follow-up data. A central finding of this review concerns the role of caregivers as active mediators of the intervention process. The effectiveness of telerehabilitation appears to be closely linked to the extent to which intervention strategies can be embedded within naturalistic family routines and supported by active caregiver involvement. Across studies, particularly those targeting challenging behaviours and communication skills, the most consistent improvements were observed in interventions that explicitly trained and involved caregivers in the delivery of therapeutic strategies. This suggests that, in the context of ID, telerehabilitation may be most effective when it enhances the caregiver’s ability to scaffold the child’s behaviour and learning within everyday routines. In this sense, the therapeutic process extends beyond the digital interface and becomes embedded in the relational and environmental context in which the child develops. At the same time, this reliance on caregiver mediation may also contribute to variability in outcomes, depending on factors such as caregiver engagement, skills, and contextual resources. In contrast, interventions primarily based on computerized cognitive training appear to follow a different pattern. While these programmes consistently demonstrate high feasibility and adherence, their effects tend to be limited to specific trained domains, with little evidence of transfer to broader cognitive or functional outcomes. This raises the possibility that observed improvements may be driven, at least in part, by repeated task exposure rather than by more generalized changes in underlying cognitive processes. These findings highlight the importance of integrating cognitive training within more ecologically valid and relationally mediated intervention frameworks, particularly for individuals with intellectual disability. Telerehabilitation should be a complementary care model that may enhance treatment intensity, ecological validity, and family participation. In particular, telehealth approaches may facilitate the integration of intervention strategies into daily routines, increasing opportunities for practice and generalization in natural environments. This aspect may be especially relevant for children and adolescents with ID, whose learning often depends on repeated contextualized experiences and caregiver support.

The review also highlights that the majority of studies were conducted in Western countries, with approximately 43% originating from the United States, indicating a geographical concentration of research activity. Interventions for individuals with ID follow a life-span framework, progressing from early motor, communication, and self-regulation skills (0–3 years) to school-readiness abilities (3–6 years), and eventually to functional academic, independent-living, and pre-vocational skills during later developmental stages (6–18 years). A complementary functional approach focuses on essential daily-life competences, including self-care, motor and language abilities, social interaction, and practical conceptual learning, and is most effective when delivered through both child-centered and family-centered practices (54). With regard to language and communication, telerehabilitation has been applied with encouraging results. Individuals with Fragile X syndrome reported improvements in engagement and inferential language use (41, 44), with limitations in relation to spontaneous language production and the generalization of gains across contexts. Studies involving participants with Prader–Willi syndrome suggest that the active involvement of children within parent-mediated training is critical for achieving improvements in play and social skills and social-cognitive functioning (30, 31). Additionally, emerging online language interventions, including music-based approaches, appear feasible and well accepted by families, although their effectiveness may depend on baseline language abilities (32). In the domain of emotional, relational, and behavioural regulation, the current literature provides limited and heterogeneous evidence regarding the effectiveness of telepsychology-based interventions. Cognitive-oriented programs, such as “The Growth Factory” (45, 46), have demonstrated improvements in mental health and self-esteem, with more pronounced effects in girls. Nonetheless, their impact appears more modest in other domains, including empowerment and treatment motivation. Telerehabilitation has also been shown to be effective in reducing challenging behaviours in children with developmental delay and Fragile X syndrome, particularly when parents are actively involved in the intervention process, with evidence of sustained effects over time (35, 36). Recent studies reinforce the central role of parent-implemented telehealth interventions, with significant reductions in parental stress and perceived challenging behaviours, alongside increased parental competence, even after brief intervention protocols (42). Furthermore, telehealth-delivered behavioural interventions such as iPCIT appear to extend their benefits to related domains, including sleep, with improvements mediated by reductions in bedtime resistance behaviours (47). Regarding executive functions, several studies have reported the feasibility and effectiveness of digital cognitive training programmes targeting attention and visuospatial and auditory working memory (49–53). These interventions were associated with high adherence rates and the maintenance of selected gains at short-term follow-up (up to three months). However, at least one study reported that improvements were not sustained at one-year follow-up, suggesting that training may need to be intensive and repeated over time to ensure long-term benefits (53). Importantly, such programmes can be effectively delivered not only by professionals but also by trained parents or teachers, supporting their scalability in home and school contexts. Recent evidence also highlights the improvement of family-assisted online interventions to promote early reading skills, with improvements generalizing across settings and maintained over time, further supporting the role of caregivers in digital rehabilitation programmes (40).

Methodological limitations remain a central concern. As highlighted by Oudshoorn et al. (55), telerehabilitation interventions in ID are characterized by substantial heterogeneity in terms of theoretical frameworks, delivery formats (synchronous vs. asynchronous), variability in intervention intensity, limited long-term follow-up, settings, participant age ranges, levels of disability, targeted outcomes, and challenges in ensuring implementation fidelity. Most interventions are individually delivered, frequently grounded in behavioural or cognitive–behavioural approaches, and implemented across diverse environments, including home, school, and residential settings. Consistent with previous reviews, the overall methodological quality of the available studies remains moderate to low (55–59). Current evidence suggests that synchronous teleconsultation with parents and caregivers represents a particularly promising approach for planning and implementing interventions aimed at behavioural management and cognitive development in children and adolescents with ID. Such models may be particularly useful for families living in underserved or rural areas, for individuals requiring high-frequency support, or for children with behavioural difficulties that limit access to traditional services. These programmes tend to be more effective when caregivers are actively involved through structured coaching, feedback, and the use of digital learning platforms. Moreover, caregiver-mediated telehealth approaches may contribute to increasing parental competence and self-efficacy, potentially improving the continuity of intervention beyond scheduled therapy sessions. In line with this, recent tele-coaching approaches have shown that remotely delivered, structured physical activity programmes can improve motor outcomes such as balance and cardiovascular indicators, while reducing access barriers for adolescents with Down syndrome (33). Similarly, remotely delivered lifestyle interventions targeting physical activity and nutrition literacy show promising feasibility and acceptability, with preliminary improvements in motor competence, motivation, and health-related behaviours (28). In practice, telerehabilitation may increase the frequency and intensity of intervention delivery, particularly during critical developmental windows and in contexts where in-person services are limited. It also facilitates interprofessional collaboration among clinicians and service providers (60–62). Guidelines emphasize the importance of clearly defining session structure, task complexity, and duration, typically not exceeding 30 minutes per session, as well as establishing individualized, SMART (Specific, Measurable, Achievable, Relevant, and Time-bound) therapeutic goals that reflect both child and family needs (63, 64). Several important gaps remain in the current evidence. Future large-scale studies are needed to determine the optimal intensity and duration of training, identify which individual profiles benefit most, clarify the transfer of gains across cognitive, academic, and social domains, and assess the long-term stability of outcomes. Emphasis should be placed on examining the extent to which trained skills generalize to ecologically valid contexts and are maintained over time, as these dimensions are critical for evaluating the clinical and functional significance of the intervention. Moreover, greater attention should be paid to the therapeutic alliance in remote settings and to the development of specific training for practitioners delivering tele-based interventions (65). Finally, the digital divide remains a critical barrier, particularly for individuals with ID who may face sensory, cognitive, and motor challenges, limited access to adaptive devices, and reduced technological literacy. Caregivers also bear a substantial burden in managing devices, troubleshooting technical issues, and ensuring safe and appropriate use of digital platforms (66). Consequently, the selection of interventions and technologies should be guided by careful user profiling, structured caregiver training, and the adoption of hybrid models that combine in-person assessment and coaching with structured synchronous and asynchronous remote interventions (61, 64, 67).

Several methodological limitations should be considered when interpreting the findings of this review. First, the included studies were highly heterogeneous in terms of intervention protocols, outcome measures, and study designs, limiting the comparability of results, variable methodological quality and precluding quantitative synthesis. Second, many studies were characterized by small sample sizes and, in some cases, limited reporting of participant characteristics, which may affect the generalizability of the findings. Third, follow-up data were often scarce or absent, making it difficult to determine the sustainability of the observed effects over time. Finally, variability in the level of caregiver involvement and in the contextual conditions under which interventions were delivered may have contributed to differences in outcomes across studies, highlighting the need for more standardized reporting and the consideration of contextual factors in future research.

## 5 Conclusions

The telerehabilitation and digital psychological interventions may provide additional opportunities for clinicians and service providers working with children and adolescents with ID and their families to support cognitive, emotional, and behavioural outcomes. These approaches may facilitate multidisciplinary collaboration, enhance participation (e.g., by reducing travel time and costs), and potentially mitigate stigma-related concerns associated with attending rehabilitation services (55). However, feasibility is likely to vary across settings and individual circumstances, indicating the need for more individualized intervention planning and greater attention to parent and caregiver involvement. The heterogeneity of intervention protocols and methodology remain key challenges. To optimize outcomes, hybrid care models combining in-person and remote components should be implemented, taking into account the context and the specific needs of each child–caregiver dyad.

## Data Availability

The original contributions presented in the study are included in the article/**Supplementary Material**. Further inquiries can be directed to the corresponding author.
