# Supplementary material for: Telerehabilitation in children and adolescents with intellectual disability: a systematic review
Source: Front Psychiatry. 2026 Jun 11;17:1855260. doi: 10.3389/fpsyt.2026.1855260 (PMC13294227; doi:10.3389/fpsyt.2026.1855260)
Supplement: Supplementary file 1 [file Table1.docx]

Supplementary Material

# eTable 1. PRSIMA 2020 Checklist

| **Section and Topic** | **Item #** | **Checklist item** | **Location where item is reported (page)** |
| --- | --- | --- | --- |
| **TITLE** | | |  |
| Title | 1 | Identify the report as a systematic review. | Title |
| **ABSTRACT** | | |  |
| Abstract | 2 | See the PRISMA 2020 for Abstracts checklist. | Abstract |
| **INTRODUCTION** | | |  |
| Rationale | 3 | Describe the rationale for the review in the context of existing knowledge. | Introduction |
| Objectives | 4 | Provide an explicit statement of the objective(s) or question(s) the review addresses. | Introduction |
| **METHODS** | | |  |
| Eligibility criteria | 5 | Specify the inclusion and exclusion criteria for the review and how studies were grouped for the syntheses. | Materials and methods – Literature search |
| Information sources | 6 | Specify all databases, registers, websites, organisations, reference lists and other sources searched or consulted to identify studies. Specify the date when each source was last searched or consulted. | Materials and methods – Literature search |
| Search strategy | 7 | Present the full search strategies for all databases, registers and websites, including any filters and limits used. | Supplemetary Materials – eMethods 1 |
| Selection process | 8 | Specify the methods used to decide whether a study met the inclusion criteria of the review, including how many reviewers screened each record and each report retrieved, whether they worked independently, and if applicable, details of automation tools used in the process. | Materials and methods – Selection process |
| Data collection process | 9 | Specify the methods used to collect data from reports, including how many reviewers collected data from each report, whether they worked independently, any processes for obtaining or confirming data from study investigators, and if applicable, details of automation tools used in the process. | Materials and methods – Data collection process |
| Data items | 10a | List and define all outcomes for which data were sought. Specify whether all results that were compatible with each outcome domain in each study were sought (e.g. for all measures, time points, analyses), and if not, the methods used to decide which results to collect. | Materials and methods – Data collection process |
|  | 10b | List and define all other variables for which data were sought (e.g. participant and intervention characteristics, funding sources). Describe any assumptions made about any missing or unclear information. | Materials and methods – Data collection process |
| Study risk of bias assessment | 11 | Specify the methods used to assess risk of bias in the included studies, including details of the tool(s) used, how many reviewers assessed each study and whether they worked independently, and if applicable, details of automation tools used in the process. | Materials and methods – Study quality; Supplementary Materials – eTable 2 |
| Effect measures | 12 | Specify for each outcome the effect measure(s) (e.g. risk ratio, mean difference) used in the synthesis or presentation of results. | Materials and methods – Data collection process (narrative synthesis; no standardized effect measures applied) |
| Synthesis methods | 13a | Describe the processes used to decide which studies were eligible for each synthesis (e.g. tabulating the study intervention characteristics and comparing against the planned groups for each synthesis (item #5)). | Materials and methods – Data collection process; Supplementary Materials – eTable 2 |
|  | 13b | Describe any methods required to prepare the data for presentation or synthesis, such as handling of missing summary statistics, or data conversions. | Materials and methods – Data collection process |
|  | 13c | Describe any methods used to tabulate or visually display results of individual studies and syntheses. | Materials and methods – Data collection process; Results – Target domains of telerehabilitation; Table 1; Figures 2–3 |
|  | 13d | Describe any methods used to synthesize results and provide a rationale for the choice(s). If meta-analysis was performed, describe the model(s), method(s) to identify the presence and extent of statistical heterogeneity, and software package(s) used. | Materials and methods – Data collection process |
|  | 13e | Describe any methods used to explore possible causes of heterogeneity among study results (e.g. subgroup analysis, meta-regression). | Not performed |
|  | 13f | Describe any sensitivity analyses conducted to assess robustness of the synthesized results. | Not performed |
| Reporting bias assessment | 14 | Describe any methods used to assess risk of bias due to missing results in a synthesis (arising from reporting biases). | Not assessed |
| Certainty assessment | 15 | Describe any methods used to assess certainty (or confidence) in the body of evidence for an outcome. | Not assessed |
| **RESULTS** | | |  |
| Study selection | 16a | Describe the results of the search and selection process, from the number of records identified in the search to the number of studies included in the review, ideally using a flow diagram. | Results – Study selection; Figure 1 |
|  | 16b | Cite studies that might appear to meet the inclusion criteria, but which were excluded, and explain why they were excluded. | Results – Study selection |
| Study characteristics | 17 | Cite each included study and present its characteristics. | Results – Study and population characteristics; Table 1; Supplementary Materials – eTable 2 |
| Risk of bias in studies | 18 | Present assessments of risk of bias for each included study. | Results – Quality assessment; Supplementary Materials – eTable 2 |
| Results of individual studies | 19 | For all outcomes, present, for each study: (a) summary statistics for each group (where appropriate) and (b) an effect estimate and its precision (e.g. confidence/credible interval), ideally using structured tables or plots. | Results – Target domains of telerehabilitation; Table 1 |
| Results of syntheses | 20a | For each synthesis, briefly summarise the characteristics and risk of bias among contributing studies. | Results – Quality assessment; Results – Target domains of telerehabilitation |
|  | 20b | Present results of all statistical syntheses conducted. If meta-analysis was done, present for each the summary estimate and its precision (e.g. confidence/credible interval) and measures of statistical heterogeneity. If comparing groups, describe the direction of the effect. | Not applicable |
|  | 20c | Present results of all investigations of possible causes of heterogeneity among study results. | Not performed |
|  | 20d | Present results of all sensitivity analyses conducted to assess the robustness of the synthesized results. | Not applicable |
| Reporting biases | 21 | Present assessments of risk of bias due to missing results (arising from reporting biases) for each synthesis assessed. | Not assessed |
| Certainty of evidence | 22 | Present assessments of certainty (or confidence) in the body of evidence for each outcome assessed. | Not assessed |
| **DISCUSSION** | | |  |
| Discussion | 23a | Provide a general interpretation of the results in the context of other evidence. | Discussion |
|  | 23b | Discuss any limitations of the evidence included in the review. | Discussion |
|  | 23c | Discuss any limitations of the review processes used. | Discussion |
|  | 23d | Discuss implications of the results for practice, policy, and future research. | Discussion |
| **OTHER INFORMATION** | | |  |
| Registration and protocol | 24a | Provide registration information for the review, including register name and registration number, or state that the review was not registered. | Introduction; Materials and methods (PROSPERO CRD420251005874) |
|  | 24b | Indicate where the review protocol can be accessed, or state that a protocol was not prepared. | Introduction; Materials and methods |
|  | 24c | Describe and explain any amendments to information provided at registration or in the protocol. | Materials and methods |
| Support | 25 | Describe sources of financial or non-financial support for the review, and the role of the funders or sponsors in the review. | Funding statement |
| Competing interests | 26 | Declare any competing interests of review authors. | Conflict of interest statement |
| Availability of data, code and other materials | 27 | Report which of the following are publicly available and where they can be found: template data collection forms; data extracted from included studies; data used for all analyses; analytic code; any other materials used in the review. | Data availability statement |

# eMethod 1. Search strategy used to search each database.

A **combination of free text search and subject heading search** was conducted using terms related to **inteleectual disability** as well as terms related to telerehabilitation. The search was limited to **studies involving humans.**

**PubMed**

("Intellectual Disability"[Mesh] OR “intellectual impairment*” OR “intellectual disabilit*” OR “intellectual dysfunction*” OR "Developmental Disability"[Mesh] OR “developmental disability*” OR “intellectual developmental disorder” OR “intellectual developmental disorder*” OR “mental deficienc*” OR “mental* retard*” OR “mental* handicap*” OR “mental* disab*” OR “mental insufficiency” OR “mental* impair*” OR “mental* subnormality” OR "Learning Disability"[Mesh] OR “learning disability*” OR “developmental* delay*” OR “developal* disab*” OR “intellect* challeng*” OR “intellect* disab*” OR “down* syndrome” OR “Rett syndrome” OR “Prader-Willy syndrome” OR “X-fragile syndrome”) AND (“Child”[Mesh] OR ”Adolescent”[Mesh]) AND ("Telerehabilitation"[Mesh] OR "Telemedicine"[Mesh] OR "telerehabilitat*" OR "tele rehabilitation" OR "Tele-rehabilitation" OR "tele health" OR "telehealth" OR "remote consultation" OR "Telepatholog*" OR ”Mobile Applications”[Mesh] OR "eHealth" OR "technology"[Mesh] OR "Videoconferencing"[Mesh] OR "videoconf*" OR ”augmented reality”[Mesh] OR "interactive multimedia" OR "interactive software" OR "digital media" OR "software"[Mesh] OR "interactive technolog*" OR "mHealth technolog*" OR "mHealth" OR "m-health" OR "mobile tech*" OR ”Video Games”[Mesh] OR "real-time monitoring device" OR ”Virtual Reality”[Mesh] OR "robotics"[Mesh] OR "Internet" OR "telephone" OR "mobile health" OR "web-based" OR "online services" OR "Online" OR "Remote Consultation*" OR "Patient portal*" OR "Computer-Assisted Instruction" OR "Website" OR "teleintervention" OR "Tele-intervention*" OR "Tele-education" OR "Telecare" OR "Teletreatment*" OR "Teletraining*" OR "Telecoaching" OR "telemonitor*" OR "Teleassessment*" OR "Telepractic*" OR "teletherap*" OR "telediagnostic*" OR "teleconference*" OR "web portal*" OR "web-deliver*" OR "web conferenc*" OR Skype* OR iChat*)

**Web of Science**

("Intellectual Disability" OR "intellectual impairment*" OR "intellectual disabilit*" OR "intellectual dysfunction*" OR "Developmental Disability" OR "developmental disability*" OR "intellectual developmental disorder" OR "intellectual developmental disorder*" OR "mental deficienc*" OR "mental* retard*" OR "mental* handicap*" OR "mental* disab*" OR "mental insufficiency" OR "mental* impair*" OR "mental* subnormality" OR "Learning Disability" OR "learning disability*" OR "developmental* delay*" OR "developal* disab*" OR "intellect* challeng*" OR "intellect* disab*" OR "down* syndrome" OR "Rett syndrome" OR "Prader-Willy syndrome" OR "X-fragile syndrome" ) AND (Child OR Adolescent) AND (Telerehabilitation OR Telemedicine OR telerehabilitat* OR "tele rehabilitation" OR Tele-rehabilitation OR "tele health" OR telehealth OR "remote consultation" OR Telepatholog* OR "Mobile Applications" OR eHealth OR technology OR Videoconferencing OR videoconf* OR "augmented reality" OR "interactive multimedia" OR "interactive software" OR "digital media" OR software OR "interactive technolog*" OR "mHealth technolog*" OR mHealth OR m-health OR "mobile tech*" OR "Video Games" OR "real-time monitoring device" OR "Virtual Reality" OR robotics OR Internet OR telephone OR "mobile health" OR web-based OR "online services" OR Online OR "Remote Consultation*" OR "Patient portal*" OR "Computer-Assisted Instruction" OR Website OR teleintervention OR Tele-intervention* OR Tele-education OR Telecare OR Teletreatment* OR Teletraining* OR Telecoaching OR telemonitor* OR Teleassessment* OR Telepractic* OR teletherap* OR telediagnostic* OR teleconference* OR "web portal*" OR web-deliver* OR "web conferenc*" OR Skype* OR iChat*)

# eFigure1. Number of studies published each year from 2012 to 2026

# eTable1. Characteristics of the included studies

| **Author (Year)** | **Country** | **MMAT study classification** | **Intervention** | **Sample (N exp/N ctrl)** | **Mean age (SD) [range] in years** | **Diagnosis** | **Technology** | **Operator** | **Results** |
| --- | --- | --- | --- | --- | --- | --- | --- | --- | --- |
| Bagner (2023) | USA | Quantitative RCT | Internet-delivered parent-child interaction therapy (iPCIT) | 75 (iPCIT)/75 referrals as usual | 3.02 (0.08) | Developmental delay with externalizing behavior problems | Encrypted videoconferencing platform; tablet computers (with data plans if needed); webcam; wireless earpiece worn by caregiver for live coaching | Remote trained therapist; caregiver under real-time coaching | Compared with referrals as usual, iPCIT improved child externalizing behavior **with moderate between-group effects at postintervention (d = 0.48), 6-month follow-up (d = 0.49), and 12-month follow-up (d = 0.50)**, child compliance, and parenting practices. **Clinically significant improvement was more frequent in the iPCIT group at postintervention (74% vs 42%, p < .001) and 6-month follow-up (73% vs 45%, p = .002), but not at 12-month follow-up (68% vs 65%, p = .85).** Caregiver stress was not significantly reduced compared with referrals as usual. |
| Bompard (2021) | Italy | Quantitative non-randomized | Home-based music therapy | 12/0 | 7.5 | Children with developmental disorders / developmental delay secondary to neurological conditions | Personalized visual soundtrack: 5-minute audio + mp4 video created from prior sessions; delivered to parents to play on TV or tablet | Parents VS composer/music-therapy team | Significant improvements were observed in sleep outcomes, including SDSC total score **(p = .010)**, sleep breathing disorders **(p = .012)**, and sleep–wake transition disorders **(p = .032)**. Parental stress decreased, with significant reductions in parental distress **(p = .001)** and defensive responding **(p = .012)**, while the PSI-SF total score did not change significantly **(p = .254)**. |
| Curtin (2024) | USA | Quantitative non-randomized | Physical and food literacy promotion program | 6/0 | 15.3 (0.8) [12–16] | Down syndrome, DeGeorge syndrome, chromosomal micro-deletion, unspecified ID | Videoconferencing platform; Tablets; instructional videos; sports and food-preparation kits mailed to homes | Trained instructors led physical activity; registered dietitian/nutrition student led food literacy; parents assisted with setup and materials at home | Small improvements were observed in motor competence **(overall** Test of Gross Motor Development-3 **Cohen’s d = 0.31; ball skills d = 0.49)**, nutrition knowledge, including food group categorization **(64.7% to 79.3% correct; Cohen’s h = 0.329)** and identification of healthier meals **(d = 0.645)** and drinks **(d = 0.462)**, as well as healthy eating behaviors, with increased willingness to eat vegetables and increased water and vegetable consumption post-intervention. |
| Dimitropoulos (2021) | USA | Quantitative non-randomized | Play-based intervention | 15/0 | 8.82 (2.03) | Prader-Willi Syndrome | Videoconferencing platform; Webcam; standardized toy set; parent manual and technology instructions | Trained interventionists; parents supported practice at home | Significant pre–post improvements were observed in imagination **(p = .01, d = 0.73)**, affect variety **(p = .04, d = 0.58)**, thematic frequency **(p = .03, d = 0.64)**, and overall fluency **(p = .001)**; organization showed a positive trend **(p = .10, d = 0.45)**. Baseline play skills were lower than the typically developing reference sample, and post-intervention fluency exceeded the typically developing mean. |
| Dimitropoulos (2022) | USA | Quantitative non-randomized | PRETEND-preschool program | Study 1: 15 (INV)/ 15 WL;  Study 2: 10 (INV)/10 WL | Study 1: 4.34 (0.93); Study 2: 3.70 (0.73) | PWS (genetic subtypes: maternal uniparental disomy [mUPD] and paternal deletion [DEL]) | Videoconferencing software; Dell Bluetooth earpiece; parent manual and digital materials; standardized toy | Trained doctoral-level interventionists; parents at home | No significant benefits were observed in Study 1, with no significant group-by-time effects for imagination **(p = .390)**, organization **(p = .279)**, affect frequency **(p = .347)**, affect variety **(p = .336)**, no play **(p = .496)**, functional play **(p = .636)**, or symbolic play **(p = .462)**. In Study 2, no significant overall group-by-time effects were found, but a significant interaction between group, genetic subtype, and time emerged for organization **(p = .046)**. Within the mUPD subtype, the intervention group improved significantly in organization **(p = .049)**, with broader descriptive gains in imagination, affect frequency, functional play, and symbolic play, whereas the DEL subtype showed minimal response. |
| Dimitropoulos (2024) | USA | Quantitative non-randomized | PRETEND- Program | 10/9 WL | 7.68 (1.00) [6–10] | PWS; neurodevelopmental genetic disorder with social-cognitive and adaptive impairments | Videoconferencing platform for remote play-based intervention and parent coaching; online survey tools for caregiver-reported measures; video recording for APS coding | Trained interventionists; Parents/caregivers | Intervention significantly improved play organization (**p = .021**), with positive trends in affect expression (**p = .100**), thematic frequency (**p = .065**), and positive interpersonal interactions (**p = .087**). Fully remote play-based telehealth was feasible and well accepted, with minimal technical difficulties and high family engagement. |
| Frizelle (2024) | Ireland | Mixed methods study | Language through music  intervention | Low dose: 35/High dose: 33 | 1.0-3.5 | Down syndrome | Videoconferencing platform, songs/music, key word signing, interactive activities | Parents/caregivers at home | Both groups showed improvements across all Down Syndrome Education outcomes over 12 weeks. **However, no significant dose effect emerged: high-dose intervention did not provide additional benefit over low-dose intervention for understanding/signing (OR = 0.92, 95% CI 0.54–1.57, p = .758), word imitation (OR = 1.04, 95% CI 0.63–1.71, p = .885), or spontaneous word use (OR = 1.02, 95% CI 0.63–1.64, p = .941). Baseline performance significantly predicted all outcomes (all p < .001).** |
| Giuriato (2025) | Italy | Quantitative non-randomized | Training programme based on  games | 18/0 | 13.7 (2.34) [9–17] | Down syndrome | Online tele-coaching platform delivering game-based, remote exercise sessions via tablet/PC | Trained sports science coaches; parents/caregivers support at home | **Walking backward balance scores improved significantly from pre- to post-intervention (1.41 ± 2.60 to 5.70 ± 4.37; p = .002), and systolic blood pressure decreased significantly (110 ± 10.1 to 97.9 ± 9.9 mmHg; p = .04). No significant changes were found for cardiorespiratory fitness (6MWT: p = .119), lower-body strength (standing broad jump: p = .609), executive function (p = .787), memory (p = .076), or language (p = .190).** Remote home-based delivery was feasible with good adherence. |
| Grenier-Martin (2022) | Canada | Quantitative RCT | Parent training for managing problem behaviors | 16/13 WL | 4.1 (1.7) [1.11–7.9] | ID and DD (chromosomal/genetic syndromes, ID, cerebral palsy) | Asynchronous online training platform; downloadable written guide; web access via PC/tablet | Parents/caregiver; PhD psychology students/researchers | Compared with the waitlist control group, the intervention significantly reduced problem behavior frequency **(p < .001, partial η² = 0.17)** and severity **(p < .05, partial η² = 0.15)**, and lowered parental stress **(p < .001, partial η² = 0.23)**. Across all participants, improvements in behavior frequency **(p < .001)**, behavior severity **(p < .001)**, and parental stress **(p < .01)** were maintained at follow-up. Parent self-efficacy increased significantly over time **(p < .01)**, and acceptability and satisfaction were high. |
| Hall (2020) | USA | Quantitative RCT | FCT | 30/27 TAU | 6.8 (2.4) [3.2–10.7] | FXS | Videoconferencing platform; iPad tablets; Bluetooth/wireless earpiece, tablet, procedural manual, sessions video-recorded | BCBAs; caregivers/parents | Compared with TAU, telehealth functional communication training **(FCT)** led to greater reductions in irritability **(p < .001, d = 0.65)**, stereotypic behavior **(p = .042, d = 0.34)**, and hyperactivity/noncompliance **(p = .005, d = 0.58)**. In-session problem behavior decreased markedly from baseline, with a mean reduction of **91.7%** by the end of treatment. Caregivers reported high acceptability, and parenting stress decreased, particularly in the child domain **(p = .013, d = 0.49)**. |
| Hall (2022) | USA | Quantitative non-randomized and quantitative RCT | FCT | 16/18 TAU | 6.68 (2.22)/7.21 (2.19) TAU | FXS | Videoconferencing platform; tablet/computer/smartphone; Bluetooth/wireless earpiece for live caregiver coaching; Cloud recording and storage | BCBAs; caregivers/parents | At mean 3-year follow-up, the telehealth FCT group showed greater reductions than TAU in irritability **(p = .001, d = −0.23)**, lethargy/social withdrawal **(p = .022, d = −0.15)**, and parental stress, including child domain **(p = .032, d = −0.19)**, parent domain **(p = .024, d = −0.18)**, and total stress **(p = .008, d = −0.21)**. In the booster sub-study, **4/6 boys showed fewer daily challenging behaviours at 4 weeks**, with a median reduction from **3.5 to 2** daily behaviour forms. |
| Hessl (2019) | US/Canada | Quantitative RCT | Cogmed | 50/50 non-adaptive Cogmed | 15.28 (3.36) [8–18] | FXS; ID | Computer/tablet-based working memory training | Parent/caregiver, trained Cogmed coaches | **Significant improvements** with effects maintained at 3-month follow-up **across the full sample were observed for visual working memory (Leiter-R Spatial Memory: post-training vs baseline, p = .003; SB-5 Block Span: p < .0001), auditory working memory (Digit Span: p < .0001), distractibility (KiTAP Distractibility Errors: p = .021), cognitive flexibility (KiTAP Flexibility False Alarms: p = .001), and parent- and teacher-reported executive functioning and attention (all p values ranging from < .0001 to .048). However, no significant overall differences emerged between adaptive and non-adaptive training conditions for the primary working memory composite outcome (p = .533). Hyperactivity/impulsivity also did not significantly improve according to parent or teacher ratings (parent ratings: p = .179; teacher ratings: p = .498).** |
| Hronis (2019) | Australia | Quantitative descriptive | Fearless Me!^©^ | 21/0 | [12–18] | ID with anxiety symptoms | CBT platform (web-based program with videos, interactive exercises, text-to-speech, login access for home practice) | Registered psychologist; teachers and aides | High feasibility and acceptability were reported, with full intervention completion **(21/21 participants)** and high attendance **(M = 8.95/10 sessions)**. Anxiety decreased particularly among participants with higher baseline anxiety; teacher-rated significant reductions were observed in social anxiety **(Reliable Change Index [RCI] > 1.96 for P3, P6, P9, P19, and P21)**, generalized anxiety **(RCI > 1.96 for P13, P15, P17, and P18)**, and total anxiety **(RCI > 1.96 for P3, P17, P19, P20, and P21)**. In-session engagement was high, although homework completion was limited, with only **4/21 participants** using the online program outside sessions, and parent data were limited due to low questionnaire completion. |
| Kiewik (2017) | The Netherlands | Quantitative non-randomized | E-learning prevention program for substance use | 35/34 | 14.72 (1.14) [12–16] | Mild to Moderate ID | Web-based e-learning platform “Prepared on time” with games, videos, quizzes, avatar-guided narration; school computers | Master’s-level psychology students/researchers; teachers/school staff | Reduced negative modelling influence for alcohol from classmates/friends **(F(1,36) = 8.669, p = .006, η² = .194)** and the immediate environment **(F(1,65) = 7.919, p = .006, η² = .109)**, with no significant effects on knowledge, attitudes, subjective norms, social pressure, or intention **(all p > .05)**. |
| Kirk (2016) | Australia | Quantitative RCT | Computerised Training Attention and Learning Initiative (TALI) | 38/37 | Training 7.77 (1.62); Control 8.68 (1.86) [4–11] | ID, DD | Touch-screen tablet, adaptive difficulty, reward system, interactive guide; 4 activities targeting selective attention | Parent/guardian; research assistant | Compared with controls, the training group showed small improvements only in selective attention **(visual search errors: post-training interaction b = −1.68, p < .05, SMD = 0.24; 3-month follow-up interaction b = −1.87, p < .05, SMD = 0.26)**. No specific training effects were found for sustained attention, attentional control, or parent/teacher-rated ADHD symptoms **(all interaction effects non-significant).** Compliance was high **(90% in the attention training group vs 70% in controls met compliance criteria; session completion did not differ significantly between groups, p = .303)**. |
| Kirk (2017) | Australia | Quantitative RCT | Computerized TALI | 38/37 | Training: 7.77 (1.62)/Control: 8.68 (1.86) [4–11] | ID, DD | Touchscreen tablet | Parent/guardian; researchers | No post-training effects were found on executive functions, literacy, or behavior. **At 3-month follow-up, the attention training group showed a small but significant improvement in numeracy compared with controls (**Test of Early Mathematics Ability–Third Edition (TEMA-3)**-3: interaction b = 2.29, p < .05, d = 0.15, 95% CI 0.01–0.29). No significant group-by-time effects were found for cardinality, vocabulary, phonological abilities, parent-rated executive functioning, teacher-rated working memory, or behavioral/emotional problems.** High feasibility and compliance were reported **(90% of the training group completed ≥15/25 sessions; training frequency did not differ from controls, p = .303).** |
| Lee (2017) | Hong Kong, China | Quantitative RCT | School-based weight management  program | 63/52 | Treatment: 13.44 (2.7)/Control: 15.31 (3.3) [8–16] | Mild ID with overweight/obesity | mHealth tools: Social media groups, mobile apps, email, phone calls, messaging app, video clips, digital educational materials | School nurses, teachers, health professionals; parents | Compared with controls, the intervention group showed lower BMI-related anthropometric values, including weight **(p = .11)**, BMI **(p = .33)**, triceps skinfold thickness **(p = .15)**, and subscapular skinfold thickness **(p = .13)**, although these differences were not statistically significant. Significant improvements were found in sports knowledge **(p < .001)**, snack choice knowledge **(p = .04)**, quality of life **(p < .001)**, self-esteem **(p < .001)**, self-figure rating **(p < .001)**, and perceived body image **(p = .008)**. Trends were observed for nutrition self-efficacy **(p = .65)** and peer-interaction self-efficacy **(p = .11)**, while no clear changes were found in preference for frying **(p = .29)**. |
| Li (2024) | China | Quantitative descriptive | Family member–assisted  Online early reading intervention  program | 3/0 | [7–8] | ID | Videoconferencing platform, image-character instruction software, laptop/computer-based digital learning materials | Trained researcher/educator; family members | **Treatment effects were very high for overall early reading skills (Tau-U = 1.00 for all three participants), high to very high for form–meaning correspondence (Tau-U = 0.95, 1.00, and 1.00), and moderate to high for form–pronunciation correspondence (Tau-U = 0.95, 0.84, and 0.84). Effects generalized across materials and family members and were maintained at follow-up. Social validity was high, with family members reporting satisfaction with the intervention and willingness to continue similar online programs.** |
| McDuffie (2017) | USA | Quantitative RCT | Spoken language intervention | 10/9 TAU | Treatment: 13.92 (2.26)/ TAU: 12.46 (1.23) [10–16] | FXS | Video teleconferencing; laptops, tablets with digitized wordless picture books; Bluetooth earpiece; cloud storage; call recording software | Licensed speech-language pathologist, trained clinicians; BCBA; parents | Compared with TAU, the treatment group showed large increases in child engagement **at home (p = .001) and in clinic mother–child interactions (p = .001)**, story-related talk **at home (p < .002)**, and maternal language facilitation strategies **including WH-questions, expansions, and fill-in-the-blank prompts (all p ≤ .001)**. Children improved in lexical diversity **at home (p = .001) and in clinic mother–child interactions (p = .030)**, but not grammatical complexity; gains generalized to mother–child interactions but not to unfamiliar examiners. |
| Miranda (2025) | Chile | Quantitative descriptive | Brief telehealth interventions on parental stress and challenging behaviors | 13/0 | 12.72 (5.04) | FXS, ID with frequent comorbid autism features | Telehealth platforms for synchronous parent coaching and intervention delivery | Certified clinical neuropsychologist; Parents/caregivers | **Parental stress significantly decreased from pre- to post-intervention (PSI-SF total score: 85 [52.5–97] to 55 [27.5–90], p = .0117), as did escape-motivated behaviours (10 [4–12.5] to 3 [0.5–8.5], p = .0132) and tangible-seeking behaviours (11.69 ± 8.27 to 7.15 ± 6.56, p = .0146). No significant group-level changes were found in broader maladaptive behaviour subscales on the ABC-CFX (all p > .05). Higher parental stress was associated with greater child irritability (p = .011), lethargy/withdrawal (p = .007), stereotypy (p = .021), and hyperactivity (p = .010).** High feasibility was reported in low-access regions. |
| Murphy (2022) | Australia | Quantitative descriptive | ABRACADABRA literacy instruction | 6/0 | [8.5–12.9] | Down syndrome | ABRA Lite web application, videoconferencing, tablet with Fitzroy Readers apps; computer mouse; digital fidelity recording | Speech-language pathologist/researcher; mothers | Significant improvements in word- and passage-level reading accuracy **(word reading accuracy: F(2,10) = 11.347, p = .003; supplementary word reading measure: F(2,10) = 24.017, p < .001; passage-level reading accuracy: F(2,10) = 10.992, p = .003)**, with some gains in functional reading comprehension **(F(2,10) = 11.780, p = .002)** but no clear improvement in conventional passage-level reading comprehension **(F(2,10) = 3.033, p = .093)**. High feasibility, engagement, procedural fidelity, and family acceptability. |
| Nelson (2018) | USA | Quantitative RCT | Parent-implemented spoken language intervention | 10/9 TAU | Treatment: 13.92 (2.26); TAU: 12.26 (1.13) [10–17] | FXS | Videoconferencing; laptops, tablets with digitized wordless picture books, Bluetooth headset, cloud upload, call recorder | Licensed clinicians/ Speech-Language Pathologist and BCBAs; parents | The intervention group produced more inferential language than controls (F(1,16)=34.642, p<.001), mainly in prompted contexts (F(1,16)=13.112, p<.001), but not spontaneous contexts. Improvements were observed in character goal-related actions (p=.012), character states (p=.009), character activities (p=.007), and object state/setting inferences (p=.005). High feasibility and strong maternal uptake of facilitation strategies. |
| Pulina (2015) | Italy | Quantitative non-randomized | Parent training program for spatial-simultaneous working memory | 39/0 | 12.5 (3) [7.8– 19.1] | Down syndrome | Training software, PC | Parent-led group; expert-led group (psychologist/clinical experts) | **Significant session effects were found for passive spatial-simultaneous working memory (F(2,74) = 57.74, p < .001), active spatial-simultaneous working memory (F(2,74) = 61.16, p < .001), spatial-sequential working memory (F(2,74) = 14.35, p < .001), and visuospatial abilities/geometric puzzles (F(2,74) = 51.23, p < .001). Gains were maintained at 1-month follow-up, with no significant decline from post-test to follow-up.** Parent-led training was effective, although improvements emerged more gradually than with expert-led training. |
| Söderqvist (2012) | Norway/Sweden | Quantitative RCT | Computerized training of non-verbal reasoning and working memory | Adaptive training: 22/ non-adaptive/active control group: 19 | 9.68 (1.58) [6–12.5] | ID | Non-verbal reasoning tasks; Visuo-spatial working memory training (Cogmed platform) | Parents; teachers; trained researchers | **Greater improvements during training predicted better performance on visuo-spatial working memory (Odd One Out: F(1,34) = 6.53, p = .015), verbal working memory (word span backwards: F(1,33) = 7.58, p = .010), and language comprehension in females (Comprehension of Instructions: F(1,13) = 5.41, p = .037). However, no significant group-level transfer effects were found for reasoning abilities (Raven’s matrices: p = .669; Block Design: p = .387), and no significant effects were maintained at the 1-year follow-up.** |
| Verberg (2021) | Netherlands | Quantitative RCT | The Growth Factory | 22/ care/school as usual: 24 | Not reported separately for <15 subgroup | Mild to borderline ID (IQ in mild range 50–69 or borderline 70–85) with frequent comorbid physical and/or psychiatric problems | Interactive modules, video clips, exercises; CBT elements; avatars/role models; “saying-is-believing” tasks); emails, mobile phone messages | Trained research assistants | No subgroup-specific results were reported for participants <15 years. Overall effects showed improvements at post-test in perseverance **(B = 0.225, p < .001)**, total mental health problems **(B = −0.164, p < .001)**, internalising problems **(B = −0.161, p < .001)**, attention problems **(B = −0.167, p < .001)**, self-esteem **(B = 0.054, p < .001)**, and therapeutic alliance/collaboration **(B = 0.154, p = .006)**. Follow-up improvements were found in mindset of intelligence at 3 months **(B = 0.148, p = .010)** and 6 months **(B = 0.148, p = .006)**, and in mindset of emotion and behaviour at 6 months **(B = 0.149, p = .001)**. |
| Verberg (2022) | Netherlands | Quantitative RCT | The Growth Factory | 22/ care/school as usual: 24 | Not reported separately for <15 subgroup | Mild to borderline ID (IQ in mild range 50–69 or borderline 70–85) with frequent comorbid physical and/or psychiatric problems | Interactive modules, video clips, exercises; CBT elements; avatars/role models; “saying-is-believing” tasks); emails, mobile phone messages | Trained research assistants | No subgroup-specific results were reported for participants under 15 years. Overall findings indicate improvements in perseverance **(B = 0.225, p < .001)** and reductions in internalising **(B = −0.161, p < .001)**, attention **(B = −0.167, p < .001)**, and total mental health problems **(B = −0.164, p < .001)** following the intervention. Effects were consistent across age groups, with no moderating effect of age **(all age interaction effects p > .05)**. |
| Warner (2025) | USA | Quantitative RCT | Internet-delivered parent-child interaction therapy and sleep quality (iPCIT) | 75/ Referrals as Usual: 75 | 3.02 (0.08) [2.8-3.2] | Developmental delays, including ASD and other medical/genetic or speech/language delays | iPCIT via videoconferencing platform with Bluetooth earpiece | Trained therapists/clinicians; parents/caregivers | Compared with referral as usual, iPCIT reduced bedtime resistance after treatment, with associated improvements in sleep quality at 6-month follow-up. Treatment condition significantly predicted bedtime resistance behaviours (β = 0.17, 90% CI 0.04–0.29, p = .01), and lower bedtime resistance significantly predicted better sleep quality at follow-up (β = 0.18, 90% CI 0.03–0.33, p = .02). However, the indirect effect was small and not statistically significant (β = 0.03, 90% CI −0.35–1.29, p = .09). |
| Zyga (2018) | USA | Quantitative descriptive | PRETEND-Preschool Program | 13/0 | 4.39 (1.05)  [3–6] | PWS | Videoconferencing platform; PC with webcam/audio; electronic handouts; PowerPoint-based session materials | Trained interventionists/research staff; parents/caregivers | **Parents reported high acceptability (mean BIRS acceptability score = 5.64/6.00), perceived effectiveness (mean = 5.57/6.00), usability (mean = 4.81/6.00), and overall satisfaction (mean = 4.75/5.00). In particular, parents rated the interventionist’s knowledge and care at the maximum score (6.00/6.00), and all respondents expressed willingness to engage again in a similar telehealth intervention.** Families reported improved strategies for play engagement, emotional understanding, flexibility, and management of problem behaviours. |

# eTable2. Quality assessment using the Mixed Methods Appraisal Tool (MMAT, Hong et al., 2018)

|  | Screening question | Qualitative | | | | | Quantitative RCT | | | | | Quantitative non RCT | | | | | Quantitative descriptive | | | | | Mixed methods | | | | |
| --- | --- | --- | --- | --- | --- | --- | --- | --- | --- | --- | --- | --- | --- | --- | --- | --- | --- | --- | --- | --- | --- | --- | --- | --- | --- | --- |
|  | Screening questions | | | | | | | | | | | | | | | | | | | | | | | | | |
| **Author, year** | S.1; S.2 | 1 | 2 | 3 | 4 | 5 | 1 | 2 | 3 | 4 | 5 | 1 | 2 | 3 | 4 | 5 | 1 | 2 | 3 | 4 | 5 | 1 | 2 | 3 | 4 | 5 |
| Bagner (2023) | Yes; Yes |  |  |  |  |  | + | + | + | + | - |  |  |  |  |  |  |  |  |  |  |  |  |  |  |  |
| Bompard (2021) | Yes; Yes |  |  |  |  |  |  |  |  |  |  | - | + | - | - | + |  |  |  |  |  |  |  |  |  |  |
| Curtin (2024) | Yes; Yes |  |  |  |  |  |  |  |  |  |  | - | + | + | - | + |  |  |  |  |  |  |  |  |  |  |
| Dimitropoulos (2021) | Yes; Yes |  |  |  |  |  |  |  |  |  |  | - | + | - | + | + |  |  |  |  |  |  |  |  |  |  |
| Dimitropoulos (2022) | Yes; Yes |  |  |  |  |  |  |  |  |  |  | - | + | - | + | + |  |  |  |  |  |  |  |  |  |  |
| Dimitropoulos (2024) | Yes; Yes |  |  |  |  |  |  |  |  |  |  | - | + | - | + | + |  |  |  |  |  |  |  |  |  |  |
| Frizelle (2024) | Yes; Yes | + | + | + | + | + | - | + | - | - | - |  |  |  |  |  |  |  |  |  |  | + | + | + | + | - |
| Giuriato (2025) | Yes; Yes |  |  |  |  |  |  |  |  |  |  | - | + | ? | - | + |  |  |  |  |  |  |  |  |  |  |
| Grenier-Martin (2022) | Yes; Yes |  |  |  |  |  | + | ? | - | ? | + |  |  |  |  |  |  |  |  |  |  |  |  |  |  |  |
| Hall (2020) | Yes; Yes |  |  |  |  |  | + | + | - | - | + |  |  |  |  |  |  |  |  |  |  |  |  |  |  |  |
| Hall (2022) study 1 | Yes; Yes |  |  |  |  |  | + | + | - | - | + |  |  |  |  |  |  |  |  |  |  |  |  |  |  |  |
| Hall (2022) study 2 | Yes; Yes |  |  |  |  |  |  |  |  |  |  | - | + | + | - | + |  |  |  |  |  |  |  |  |  |  |
| Hessl (2019) | Yes; Yes |  |  |  |  |  | + | + | + | + | + |  |  |  |  |  |  |  |  |  |  |  |  |  |  |  |
| Hronis (2019) | Yes; Yes |  |  |  |  |  |  |  |  |  |  |  |  |  |  |  | - | - | + | - | + |  |  |  |  |  |
| Kiewik (2017) | Yes; Yes |  |  |  |  |  |  |  |  |  |  | - | + | + | - | + |  |  |  |  |  |  |  |  |  |  |
| Kirk (2016) | Yes; Yes |  |  |  |  |  | + | - | + | + | + |  |  |  |  |  |  |  |  |  |  |  |  |  |  |  |
| Kirk (2017) | Yes; Yes |  |  |  |  |  | + | - | + | + | + |  |  |  |  |  |  |  |  |  |  |  |  |  |  |  |
| Lee (2017) | Yes; Yes |  |  |  |  |  | + | - | + | ? | + |  |  |  |  |  |  |  |  |  |  |  |  |  |  |  |
| Li (2024) | Yes; Yes |  |  |  |  |  |  |  |  |  |  |  |  |  |  |  | - | - | + | + | + |  |  |  |  |  |
| McDuffie (2017) | Yes; Yes |  |  |  |  |  | + | + | + | ? | + |  |  |  |  |  |  |  |  |  |  |  |  |  |  |  |
| Miranda (2025) | Yes; Yes |  |  |  |  |  |  |  |  |  |  |  |  |  |  |  | - | - | + | + | + |  |  |  |  |  |
| Murphy (2022) | Yes; Yes |  |  |  |  |  |  |  |  |  |  |  |  |  |  |  | - | - | + | + | + |  |  |  |  |  |
| Nelson (2018) | Yes; Yes |  |  |  |  |  | + | + | ? | - | + |  |  |  |  |  |  |  |  |  |  |  |  |  |  |  |
| Pulina (2015) | Yes; Yes |  |  |  |  |  |  |  |  |  |  | + | + | + | - | + |  |  |  |  |  |  |  |  |  |  |
| Söderqvist (2012) | Yes; Yes |  |  |  |  |  | + | + | + | + | + |  |  |  |  |  |  |  |  |  |  |  |  |  |  |  |
| Verberg (2021) | Yes; Yes |  |  |  |  |  | + | + | - | - | + |  |  |  |  |  |  |  |  |  |  |  |  |  |  |  |
| Verberg (2022) | Yes; Yes |  |  |  |  |  | + | + | + | - | + |  |  |  |  |  |  |  |  |  |  |  |  |  |  |  |
| Warner (2025) | Yes; Yes |  |  |  |  |  | + | + | + | - | + |  |  |  |  |  |  |  |  |  |  |  |  |  |  |  |
| Zyga (2018) | Yes; Yes |  |  |  |  |  |  |  |  |  |  |  |  |  |  |  | - | - | + | ? | + |  |  |  |  |  |

**Screening questions**: S1. Are the research questions clearly stated? S2. Do the collected data allow the research questions to be addressed? **Qualitative studies items: 1**. Is the qualitative approach appropriate to answer the research question? **2.** Are the qualitative data collection methods adequate to address the research question? **3.**Are the findings adequately derived from the data? **4.** Is the interpretation of results sufficiently substantiated by data? **5.** Is there coherence between data sources, data collection, analysis, and interpretation? **Quantitative randomized controlled trials** **items**: **1.** Is randomization appropriately performed? **2.** Are the groups comparable at baseline? **3.** Are there complete outcome data? **4.** Are outcome assessors blinded to the intervention provided? **5.** Did the participants adhere to the assigned intervention? **Quantitative non-randomized studies items: 1.** Are the participants representative of the target population? **2.** Are the measurements appropriate regarding both the outcome and the intervention (or exposure)? **3.** Are there complete outcome data? **4.** Are the confounders accounted for in the design and analysis? **5.** During the study period, is the intervention administered (or exposure occurred) as intended? **Quantitative descriptive studies items: 1.** Is the sampling strategy relevant to address the research question? **2.** Is the sample representative of the target population? **3.** Are the measurements appropriate? **4.** Is the risk of nonresponse bias low? **5.** Is the statistical analysis appropriate to answer the research question? **Mixed methods studies items: 1.** Is there an adequate rationale for using a mixed methods design to address the research question? **2.** Are the different components of the study effectively integrated to answer the research question? **3.** Are the outputs of the integration of qualitative and quantitative components adequately interpreted? **4.** Are divergences and inconsistencies between quantitative and qualitative results adequately addressed? **5.** Do the different components of the study adhere to the quality criteria of each tradition of the methods involved?

**
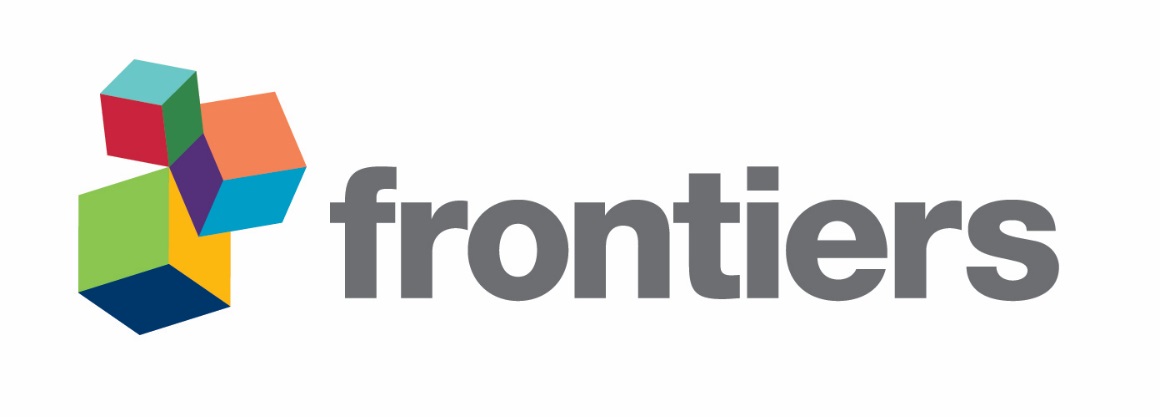
**
